# Advanced structural brain aging in preclinical autosomal dominant Alzheimer disease

**DOI:** 10.1186/s13024-023-00688-3

**Published:** 2023-12-19

**Authors:** Peter R Millar, Brian A Gordon, Julie K Wisch, Stephanie A Schultz, Tammie LS Benzinger, Carlos Cruchaga, Jason J Hassenstab, Laura Ibanez, Celeste Karch, Jorge J Llibre-Guerra, John C Morris, Richard J Perrin, Charlene Supnet-Bell, Chengjie Xiong, Ricardo F Allegri, Sarah B Berman, Jasmeer P Chhatwal, Patricio A Chrem Mendez, Gregory S Day, Anna Hofmann, Takeshi Ikeuchi, Mathias Jucker, Jae-Hong Lee, Johannes Levin, Francisco Lopera, Yoshiki Niimi, Victor J Sánchez-González, Peter R Schofield, Ana Luisa Sosa-Ortiz, Jonathan Vöglein, for the Dominantly Inherited Alzheimer Network, Randall J Bateman, Beau M Ances, Eric M McDade

**Affiliations:** 1 Department of Neurology, Washington University in St. Louis, St. Louis, MO USA; 2 Mallinckrodt Institute of Radiology, Washington University in St. Louis, St. Louis, MO USA; 3 Department of Neurology, Harvard Medical School, Boston, MA USA; 4 Department of Neurology, Massachusetts General Hospital, Boston, MA USA; 5 Department of Psychiatry, Washington University in St. Louis, St. Louis, MO USA; 6 NeuroGenomics & Informatics Center, Washington University in St. Louis, St. Louis, MO USA; 7 Department of Pathology & Immunology, Washington University in St. Louis, St. Louis, MO USA; 8 Department of Biostatistics, Washington University in St. Louis, St. Louis, MO USA; 9 Instituto Neurológico Fleni, Buenos Aires, Argentina; 10 Department of Neurology, University of Pittsburgh, Pittsburgh, PA USA; 11 Department of Neurology, Mayo Clinic, Jacksonville, FL USA; 12 German Center for Neurodegenerative Diseases (DZNE), 72076 Tübingen, Germany; 13 Department of Cellular Neurology, Hertie Institute for Clinical Brain Research, University of Tübingen, 72076 Tübingen, Germany; 14 Department of Molecular Genetics, Brain Research Institute, Niigata University, Niigata, Japan; 15 Department of Neurology, University of Ulsan College of Medicine, Asan Medical Center, Seoul, Republic of Korea; 16 Department of Neurology, Ludwig-Maximilians-Universität München, Munich, Germany; 17 German Center for Neurodegenerative Diseases, Munich, Germany; 18 Munich Cluster for Systems Neurology (SyNergy), Munich, Germany; 19 Universidad de Antioquia, Medellín, Colombia; 20 Unit for Early and Exploratory Clinical Development, The University of Tokyo Hospital, Bunkyo-Ku, Tokyo, Japan; 21 Departamento de Clínicas, CUALTOS, Universidad de Guadalajara, Tepatitlán de Morelos, Jalisco, México; 22 Neuroscience Research Australia, Sydney, NSW Australia; 23 School of Biomedical Sciences, University of New South Wales, Sydney, NSW Australia; 24 Instituto Nacional de Neurologia y Neurocirugía MVS, CDMX, Ciudad de México, Mexico

**Keywords:** Brain aging, Alzheimer disease, Structural MRI, Machine learning

## Abstract

**Background:**

“Brain-predicted age” estimates biological age from complex, nonlinear features in neuroimaging scans. The brain age gap (BAG) between predicted and chronological age is elevated in sporadic Alzheimer disease (AD), but is underexplored in autosomal dominant AD (ADAD), in which AD progression is highly predictable with minimal confounding age-related co-pathology.

**Methods:**

We modeled BAG in 257 deeply-phenotyped ADAD mutation-carriers and 179 non-carriers from the Dominantly Inherited Alzheimer Network using minimally-processed structural MRI scans. We then tested whether BAG differed as a function of mutation and cognitive status, or estimated years until symptom onset, and whether it was associated with established markers of amyloid (PiB PET, CSF amyloid-β-42/40), phosphorylated tau (CSF and plasma pTau-181), neurodegeneration (CSF and plasma neurofilament-light-chain [NfL]), and cognition (global neuropsychological composite and CDR-sum of boxes). We compared BAG to other MRI measures, and examined heterogeneity in BAG as a function of ADAD mutation variants, APOE ε4 carrier status, sex, and education.

**Results:**

Advanced brain aging was observed in mutation-carriers approximately 7 years before expected symptom onset, in line with other established structural indicators of atrophy. BAG was moderately associated with amyloid PET and strongly associated with pTau-181, NfL, and cognition in mutation-carriers. Mutation variants, sex, and years of education contributed to variability in BAG.

**Conclusions:**

We extend prior work using BAG from sporadic AD to ADAD, noting consistent results. BAG associates well with markers of pTau, neurodegeneration, and cognition, but to a lesser extent, amyloid, in ADAD. BAG may capture similar signal to established MRI measures. However, BAG offers unique benefits in simplicity of data processing and interpretation. Thus, results in this unique ADAD cohort with few age-related confounds suggest that brain aging attributable to AD neuropathology can be accurately quantified from minimally-processed MRI.

**Supplementary Information:**

The online version contains supplementary material available at 10.1186/s13024-023-00688-3.

Human biological aging is marked by complex, nonlinear changes in brain structure that can be observed in vivo using magnetic resonance imaging (MRI) [1–3]. Recent efforts to model biological aging have applied machine learning techniques to large MRI datasets of cognitively normal participants in order to capture normative trajectories of structural brain features across the lifespan [4, 5]. Critically, this approach estimates brain-predicted biological age in individual participants relative to the normative training sample. Typical brain aging is expected to produce a small brain age gap (BAG) between model-predicted brain age and true chronological age [4, 5]. When the brain-predicted age is older (BAG > 0) or younger (BAG < 0) than chronological age, these deviations are interpreted as signals of advanced or resilient biological aging, respectively.

A growing body of literature provides converging evidence that BAG is influenced by a wide range of neurological, psychiatric, and general health conditions, as well as potential resilience factors [6, 7]. Consistent elevations in brain age are reported in the symptomatic stages of Alzheimer disease (AD) [6–8]. Although AD-related atrophy can be detected using other established biomarkers from structural MRI [9], at least one study suggests that a brain age estimate offers improved detection of symptomatic AD progression even beyond established AD biomarkers, such as hippocampal volume [10]. Thus, by capturing complex, multivariate, nonlinear patterns of brain aging, this approach might reflect a more comprehensive and sensitive view of disease-related pathology and risk, above and beyond individual features derived from the same MRI scans.

Although advanced structural brain aging has been clearly established in symptomatic AD [6, 7], it is not clear whether these estimates are sensitive to the preclinical stage, i.e., the presence of amyloid-β pathology in the absence of cognitive decline [11]. While studies have consistently demonstrated associations between structural brain age estimates and AD biomarkers in symptomatic Alzheimer dementia samples [12, 13], two recent studies of BAG in cognitively unimpaired participants did not observe associations with AD biomarkers [14–16].

Thus far, most studies of brain aging in AD have focused on sporadic late onset AD (sLOAD). Another critical population for evaluating the preclinical phase is autosomal dominant AD (ADAD) [17, 18]. ADAD mutation-carriers have a highly predictable age of symptom onset, which can be used to model the timecourse of early pathological progression as a function of estimated years until symptom onset (EYO). Additionally, since ADAD samples are younger than sLOAD, observed deviations in brain age are less likely to be attributed to other age-related etiologies (e.g., cerebrovascular disease). Indeed, one recent study demonstrated that an estimate of brain age using *resting-state functional connectivity* MRI is elevated in ADAD mutation-carriers and in association with amyloid PET [19]. However, this study did not examine structural brain aging, which is more accessible in clinical and research settings and might capture complementary pathological signal to functional connectivity.

In the present study, we applied a recently developed, publicly available convolutional neural network, DeepBrainNet (DBN) [20], to model brain age in a sample of ADAD mutation-carriers (MCs) and non-carriers (NCs) from the Dominantly Inherited Alzheimer Network (DIAN) [17, 18]. Importantly, DBN has been trained on independent datasets spanning the human lifespan to predict age using minimally-processed, whole-brain structural MRI scans as inputs [20] (see Fig. 1). Thus, brain age estimates from DBN may be derived quickly and automatically, with less intensive preprocessing and quality assessments than required for other brain age models or established MRI biomarkers. With this method, we modeled the emergence of advanced structural brain aging over the course of ADAD progression as defined by EYO. To further evaluate the sensitivity of brain aging to AD pathology, we tested associations with other established PET and biofluid AD biomarkers, as well as measures of cognition. To test the additional utility of brain age estimates in capturing AD-related atrophy, we compared brain age to other established MRI biomarkers in their ability to detect divergence between MCs and NCs. Given prior demonstrations of heterogeneity in pathological burden and age of onset between ADAD mutation variants [21–24], we tested whether brain age estimates captured similar differences in pathological severity. Finally, as prior studies have demonstrated differences in brain aging as a function of sex [14, 25–29], education [30, 31], and *APOE* genotype [32], we tested those relationships in this ADAD sample.

**Fig. 1:**
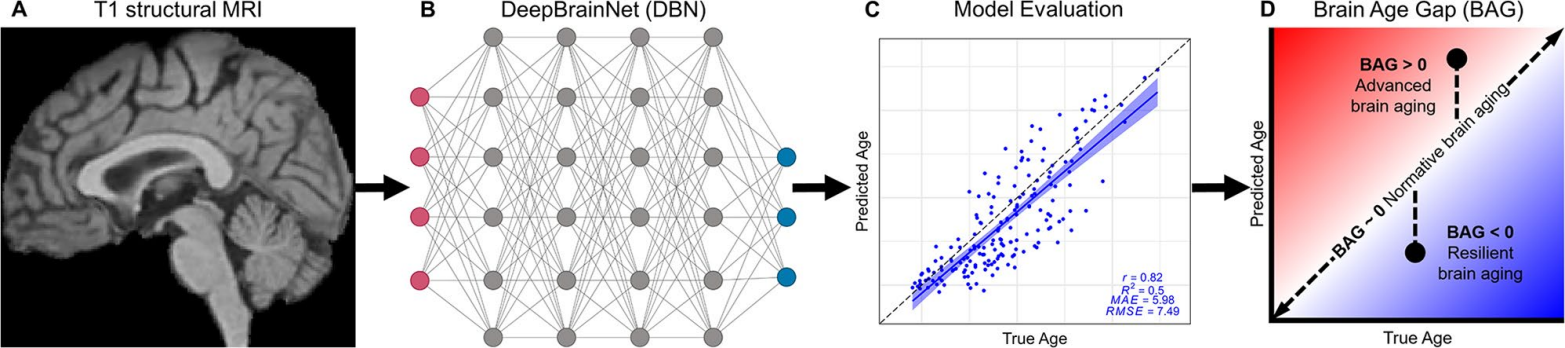
Overview of study design. (**A**) Whole-brain T1 structural MR images from DIAN were processed with brain extraction and linear registration to an atlas template, and were then used as input data for brain age analyses. (**B**) DeepBrainNet (DBN) is a publicly available convolutional neural network that has previously been trained to predict age in 11,729 MRI scans from independent control samples [20]. (**C**) Model prediction accuracy of age was evaluated by calculating Pearson’s correlation coefficient (*r*), coefficient of determination (*R*^*2*^), mean absolute error (*MAE*), and root mean square error (*RMSE*) in non-carrier participants. (**D**) Brain Age Gap (BAG) for each DIAN participant was calculated as the difference between DBN-predicted and true chronological age. Figure created in part with BioRender.com and modified from [33]

## Methods

### Participants

The sample included 436 participants from the DIAN Observational Study (data release 16) [17, 18]. 257 participants were identified as mutation-carriers (MCs) of pathologic variants in presenilin-1 (*PSEN1*), presenilin-2 (*PSEN2*), or amyloid precursor protein (*APP*). *PSEN1* mutation-carriers were further grouped based on the location of the underlying variant (i.e., before or after codon 200) [21]. The remaining 179 participants were non-carrier controls (NCs), recruited from the same families as the MCs. In order to characterize and correct for differences in brain age estimates between different sites and scanners, we included data only from DIAN sites with at least 5 eligible participants with a usable T1 MRI scan. For details on each site-specific sample, see Supplementary Table 1.

Estimated years until symptom onset (EYO) was computed for each participant as the difference between the participant’s chronological age and the mean age of symptom onset for their specific mutation, as determined by a database of known mutation onsets, or the familial age of symptom onset, as determined by semistructured interview [17, 18]. Thus, negative EYO values reflect years until expected onset, whereas positive values reflect years after onset.

Cognitive status was determined using the Clinical Dementia Rating® (CDR®) scale [34]. A CDR of 0 defines cognitive normality, while scores above 0 define increasing stages of dementia severity. MCs with CDR = 0 were identified as “asymptomatic”, while MCs with CDR > 0 were identified as “symptomatic”. *APOE* ε4 status was dichotomized in all participants between ε4 carriers (ε4+, including heterozygotes and homozygotes) and ε4 non-carriers (ε4-).

DIAN participants provided informed consent in accordance with the local institutional review boards of each participating site. DIAN study procedures have received ethics approval by the Human Research Protection Office at Washington University in St. Louis (MO, USA) and all of the participating sites.

### PET & biofluid biomarkers

Amyloid β plaque deposition was assessed using a [^11^C] Pittsburgh Compound B (PiB) PET tracer. PET data were analyzed from a 30-minute acquisition window beginning 40 min after a bolus injection of approximately 15 mCi of PiB. All PiB PET scans were processed with the PET Unified Pipeline (PUP) [35], including conventional processing steps and partial volume correction with a regional spread function (RSF). Standardized uptake value ratios (SUVR) summarized PiB tracer binding in previously-defined summary regions, including bilateral precuneus, prefrontal cortex, gyrus rectus, and lateral temporal regions, using the cerebellum as a reference region [35].

Cerebrospinal fluid (CSF) samples were collected via lumbar puncture under fasting conditions [17]. CSF amyloid β42 (Aβ42), amyloid β40 (Aβ40), and phosphorylated tau-181 (pTau) were measured with Lumipulse immunoassays (Fujirebio). Aβ42 and pTau estimates were normalized for individual differences in CSF production rates by forming a ratio with Aβ40 as the denominator [36, 37]. Neurofilament-light-chain (NfL) was measured with a Simoa HD-X platform (Quanterix).

Blood samples were collected via venipuncture under fasting conditions [38]. Plasma pTau and NfL were measured on a Simoa HD-X platform (Quanterix).

### Cognitive battery

Participants completed a comprehensive neuropsychological test battery [39]. We formed a global cognitive composite by standardizing and combining the Logical Memory delayed recall score [40], Digit Symbol Substitution total score [41], Animal Naming fluency score [42], and time to complete Trail Making part B [43].

We also used the CDR-Sum of Boxes as an additional measure of global dementia severity [44].

### MRI acquisition & processing

Structural MRI scans were acquired on a 3T scanner using a T1-weighted magnetization-prepared rapid gradient echo sequence (MPRAGE; echo time [TE] = 2.95 ms, repetition time [TR] = 2300 ms, inversion time [TI] = 900 ms, field of view [FOV] = 270 mm, flip angle = 9°, 225 slices, 1.1 × 1.1 × 1.2 mm^3^ voxels) [45]. Scanner manufacturer and model varied between DIAN sites. T1-weighted images were processed using a common minimal pipeline including brain extraction and linear registration to the MNI 152 atlas template.

T1-weighted images were also processed with FreeSurfer 5.3 [46]. For comparison with BAG, additional analyses examined structural estimates in *a priori* regions of interest, including hippocampus volume [18], precuneus thickness [47], and cortical thickness in AD-specific signature regions [48].

### Brain age modeling

DeepBrainNet (DBN) is a publicly available 2D convolutional neural network using the inception-resnet-v2 framework, which has previously been trained to predict age on 11,729 MRI scans from independent samples [20]. Briefly, minimally-processed whole-brain T1-weighted images were each represented as a collection of 80 evenly spaced axial slices, which matched the slice positioning of the original DBN training set. For each participant, each of the 80 slices were provided to DBN as independent inputs. A predicted age was generated for each slice with the median output serving as the participant’s final age prediction [20].

To correct for regression dilution in the age prediction model (see Supplementary Fig. 1) [49], we applied a linear transformation to predicted age values, adjusting for the slope and intercept from a regression model of predicted age as a function of chronological age in NCs [25, 50]. BAG was then calculated as the difference between corrected brain age and chronological age and this corrected value was used for statistical tests of group differences and associations with other variables. However, to avoid inflating estimates of prediction accuracy, only uncorrected age prediction values were used to evaluate model performance [51, 52].

### Statistical harmonization

Since MRI scans were collected across several DIAN sites, using a variety of scanner models, these differences might contribute non-biological variance to the brain age signal. Indeed, a Kruskall-Wallis test revealed that BAG values differed between NCs tested at different DIAN sites, *χ*^*2*^ (14) = 27.72, *p* = 0.015, and on different scanner models, *χ*^*2*^ (8) = 15.88, *p* = 0.044 (see Supplementary Figs. 2 and 3). Although these differences were relatively small and limited to only a few specific sites and scanners, they violated our assumption that BAG values should be centered near 0 in NCs, regardless of the site or scanner of acquisition, and had the potential to introduce unwanted noise or confound into the brain age signal.

Hence, we harmonized brain-predicted age values between sites using ComBat [53]. We included age, sex, education, ADAD group, EYO, *APOE* ε4 status, and mutation variant as covariates during harmonization to preserve variance in BAG related to these variables of interest. Critically, after harmonization with ComBat, there were no significant differences in BAG values between sites, *χ*^*2*^ (14) = 11.70, *p* = 0.63, or scanners, *χ*^*2*^ (8) = 5.11, *p* = 0.75 (see Supplementary Figs. 2 and 3).

### Statistical analysis

All statistical analyses were conducted in R 4.2.2 (R Core Team). Assumptions of normality and homogeneity of variance were tested, respectively, by evaluating quantile-quantile plots and with Levene’s test. Mutation group differences in demographic and descriptive variables between NCs and all MCs were tested with Wilcoxon rank sum tests for continuous variables and Pearson’s *χ*^*2*^ (for cell counts ≥ 5) or Fisher’s exact test (for cell counts < 5) for categorical variables. DBN prediction accuracy was evaluated by calculating Pearson’s correlation coefficient (*r*), coefficient of determination (*R*^*2*^), mean absolute error (*MAE*), and root mean square error (*RMSE*) between predicted age and true chronological age in NCs only. Test-retest reliability of MRI measures was assessed in a subset of 182 participants who had longitudinal MRI data available. See Supplementary Table 2 for a summary of these participants at the baseline visit. There was an average of 2.11 years (SD = 1.21) between longitudinal scans. Specifically, we used the ‘irr’ package to calculate the intraclass correlation coefficient (ICC) using a 2-way mixed-effects model based on a single measurement and absolute agreement [54].

Mutation group differences in BAG between NCs, asymptomatic MCs, and symptomatic MCs were tested with an omnibus Kruskal-Wallis rank sum test, and were followed up with *post hoc* Wilcoxon tests using a false discovery rate (FDR) correction for multiple comparisons.

As non-linear trajectories in AD biomarkers have been well established in ADAD [18, 47, 55], we modeled age-corrected BAG and other MRI measures as a function of EYO using generalized additive mixed models (GAMMs), similar to an approach previously applied in both ADAD and Down syndrome [56]. Specifically, we used the ‘mgcv’ package to fit GAMMs with a restricted maximum likelihood method. GAMMs included spline terms for EYO with 4 cubic basis functions to account for non-linearity and an interaction term for mutation status. GAMMs also included categorical and linear terms for covariates, including sex, education, and *APOE* ε4 positivity. All GAMMs included a random effect term to account for familial relationships between MCs and NCs. Simultaneous 83.4% confidence intervals for comparison of two groups [57, 58] were derived for GAMM fits using a simulation-based method [59] and were used to identify the earliest points (EYO) of significant differences between MCs and NCs. We tested for differences in the point of ADAD divergence between different MRI measures using a bootstrapping analysis in 10,000 randomly resampled simulations of the full dataset.

Continuous relationships between BAG and estimates of amyloid, pTau, neurodegeneration, and cognition were tested using linear mixed effects models (LMEs). LMEs included a fixed term for each biomarker of interest and a group by biomarker interaction term, as well as covariates, including sex, education, and *APOE* ε4 positivity, and a random effect term for familial relationships. Significant group by biomarker interactions were followed-up with Pearson correlation analyses within specific subgroups. As an additional sensitivity analysis to correct for skewed distributions in the biomarker values, we also repeated the biomarker association analyses after applying a log transformation. To further characterize the complex relationships between cognition and AD progression, we tested whether BAG mediated associations between AD biomarkers and the global cognitive composite, using a non-parametric bootstrap method [60].

### Data & code availability

This study utilized datasets obtained from the DIAN Observational Study (Data Freeze 16). The data are available to all qualified researchers after appropriate review. Requests for data access may be placed to the DIAN Steering Committee (https://dian.wustl.edu/our-research/for-investigators/dian-observational-study-investigator-resources/data-request-terms-and-instructions/). Code used in this study is available at https://github.com/peterrmillar/DIANBrainAge.

**Table 1:** Demographic information for the sample

	*N*	ADAD Mutation Groups			NCs vs. All MCs		
		NCs (179)	Asymptomatic MCs (183)	Symptomatic MCs (74)	*η²*		*p*-value^1^
**Age**	436				0.000		0.79
*Mean (SD)*		37.5 (10.6)	34.3 (9.1)	46.3 (8.7)			
**Sex**	436						0.75
*Female*		101 (56%)	104 (57%)	37 (50%)			
*Male*		78 (44%)	79 (43%)	37 (50%)			
**Education**	436				0.007		0.030
*Mean (SD)*		15.0 (2.8)	14.8 (2.7)	13.7 (3.1)			
**Self-Selected Race**	436						0.69
Aboriginal Australian or Torres Strait Islander*		< 5	< 5	< 5			
American Indian or Alaska Native*		< 5	< 5	< 5			
Asian*		5 (2.8%)	< 5	< 5			
Black or African American*		< 5	< 5	< 5			
Hispanic or Latinx*		< 5	< 5	< 5			
Middle Eastern or North African*		< 5	< 5	< 5			
Native Hawaiian or Other Pacific Islander*		< 5	< 5	< 5			
*White*		152 (85%)	155 (85%)	64 (86%)			
*More than one race*		14 (7.8%)	11 (6.0%)	4 (5.4%)			
*Unknown*		5 (2.8%)	10 (5.5%)	2 (2.7%)			
**CDR**	436						< 0.001
*0*		179 (100%)	183 (100%)	0 (0%)			
*0.5*		0 (0%)	0 (0%)	44 (59%)			
*1*		0 (0%)	0 (0%)	26 (35%)			
*2*		0 (0%)	0 (0%)	4 (5.4%)			
**EYO**	436				0.003		0.16
*Mean (SD)*		-11.0 (11.4)	-14.3 (9.0)	1.4 (5.7)			
**APOE**	436						0.86
*ε4-*		124 (69%)	124 (68%)	52 (70%)			
*ε4+*		55 (31%)	59 (32%)	22 (30%)			
**Variant**	436						0.45
*APP*		38 (21%)	38 (21%)	13 (18%)			
*PSEN1 Codon < 200*		55 (31%)	48 (26%)	23 (31%)			
*PSEN1 Codon 200+*		65 (36%)	75 (41%)	37 (50%)			
*PSEN2*		21 (12%)	22 (12%)	1 (1.4%)			

* Fewer than 5 participants per group selected this race. Specific numbers and percentages for these groups are not reported to prevent unblinding of participant mutation-carrier status

ADAD, Autosomal Dominant Alzheimer Disease; NC, Non-carrier; MC, Mutation-carrier; CDR, Clinical Dementia Rating; EYO, Estimated years until symptom onset; *APOE*, Apolipoprotein E; *APP*, Amyloid Precursor Protein; *PSEN*, Presenilin

^1^Wilcoxon rank sum test; Pearson’s Chi-squared test; Fisher’s exact test

## Results

### Sample description & model performance

The final sample included 257 ADAD MCs (74 symptomatic, 183 asymptomatic) and 179 NCs from the DIAN cohort (see Table 1). Overall, MCs and NCs were well matched in age, sex, self-selected race, EYO, *APOE* genotype, and ADAD mutation variants, but NCs reported a greater number of years of education than MCs, *W* = 25,774, *p* = 0.03 (see Table 1).

The DBN model accurately predicted chronological age in the independent DIAN testing set, as evaluated in the NC participants (*r* = 0.82, *R*^*2*^ = 0.50, *MAE* = 5.98, *RMSE* = 7.49; see Fig. 1C). Although these performance metrics are low compared to those observed in the original DBN training set (*r* = 0.98, *MAE* = 3.70 [20]), we note that direct comparison of these metrics is complicated by differences in the mean and range of age values between the samples (DIAN NC *M*_*Age*_ = 37.3, *Range*_*Age*_ = 18–69; DBN train *M*_*Age*_ = 47.3, *Range*_*Age*_ = 3–95 [20]). Indeed, differences in age distributions have been shown to moderate metrics of age prediction performance [52].

In a subset of 182 participants with longitudinal MRI data available, both DBN-predicted brain age (ICC = 0.94) and BAG (ICC = 0.90) achieved excellent test-retest reliability. BAG values were not normally distributed in asymptomatic MCs (see Supplementary Fig. 4) and variance in BAG differed between groups, Levene’s statistic (2, 433) = 10.67, *p* < 0.001. Hence, non-parametric tests were used to test group differences in BAG.

### BAG differences in ADAD mutation groups

**Fig. 2:**
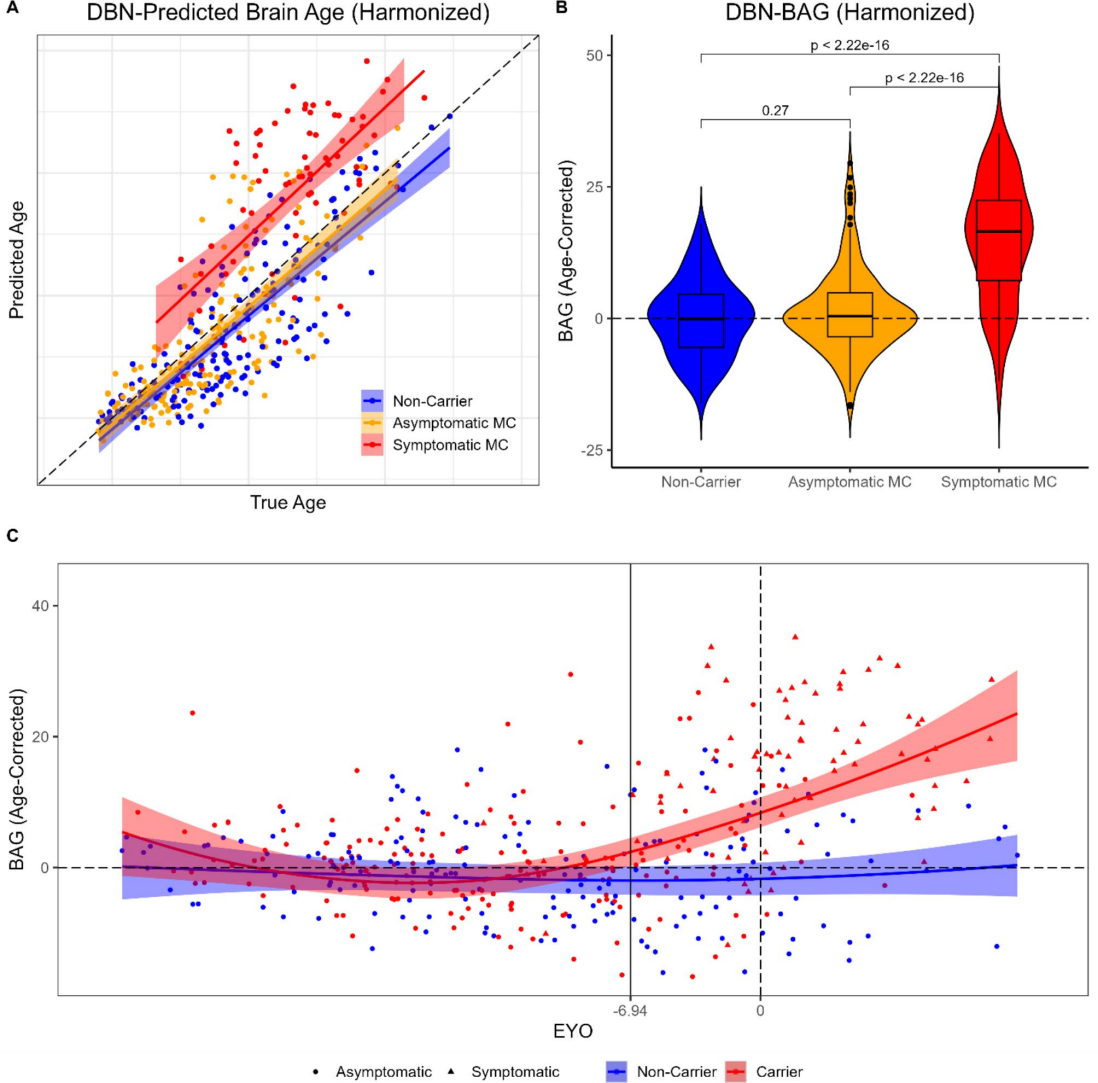
Brain age estimates as a function of ADAD mutation status and progression. (**A**) Scatterplot of DBN-predicted brain age (after harmonizing across scanner and site differences) as a function of true chronological age. Lines and shaded regions reflect regression fits and confidence intervals derived from a linear regression model. Dashed line reflects perfect age prediction. (**B**) Violin plot of brain age gap (BAG) after correcting for chronological age. Dashed line reflects perfect age prediction. *P* values are derived from Wilcoxon tests using a false discovery rate (FDR) correction. (**C**) Scatterplot of age-corrected BAG as a function of EYO. Curves and shaded regions reflect regression fits derived from a generalized additive mixed model (GAMM) and simulation-based [59] simultaneous 83.4% confidence intervals for comparison of two groups [57, 58]. Horizontal dashed line reflects perfect age prediction. Vertical dashed line reflects EYO of 0. Vertical solid line reflects the earliest EYO at which MCs significantly differed from NCs, based on the simultaneous confidence intervals. Axis labels and outlier points have been censored to prevent unblinding of participant mutation-carrier status

An omnibus Kruskal-Wallis test identified significant differences in BAG between NCs, asymptomatic MCs, and symptomatic MCs, *χ*^*2*^ (2) = 86.49, *p* < 0.001. Follow-up Wilcoxon tests indicated that BAG was greater in symptomatic MCs than in NCs (*p*_*FDR*_ < 0.001) or asymptomatic MCs (*p*_*FDR*_ < 0.001), see Fig. 2A & B. BAG did not significantly differ between asymptomatic MCs and NCs (*p*_*FDR*_ = 0.27).

A GAMM identified no evidence of a relationship between age-corrected BAG and EYO in NCs, controlling for sex, education, and *APOE*, *EDF* = 1.89, *p* = 0.43. Mutation status interacted with the spline EYO term, indicating a significant nonlinear association between BAG and EYO in MCs, *EDF* = 2.71, *p* < 0.001. Examination of overlapping confidence intervals indicated that significant differences in BAG between MCs and NCs were apparent about 7 years before expected symptom onset (-6.94 EYO; see Fig. 2C).

### Relationships with amyloid markers

**Fig. 3:**
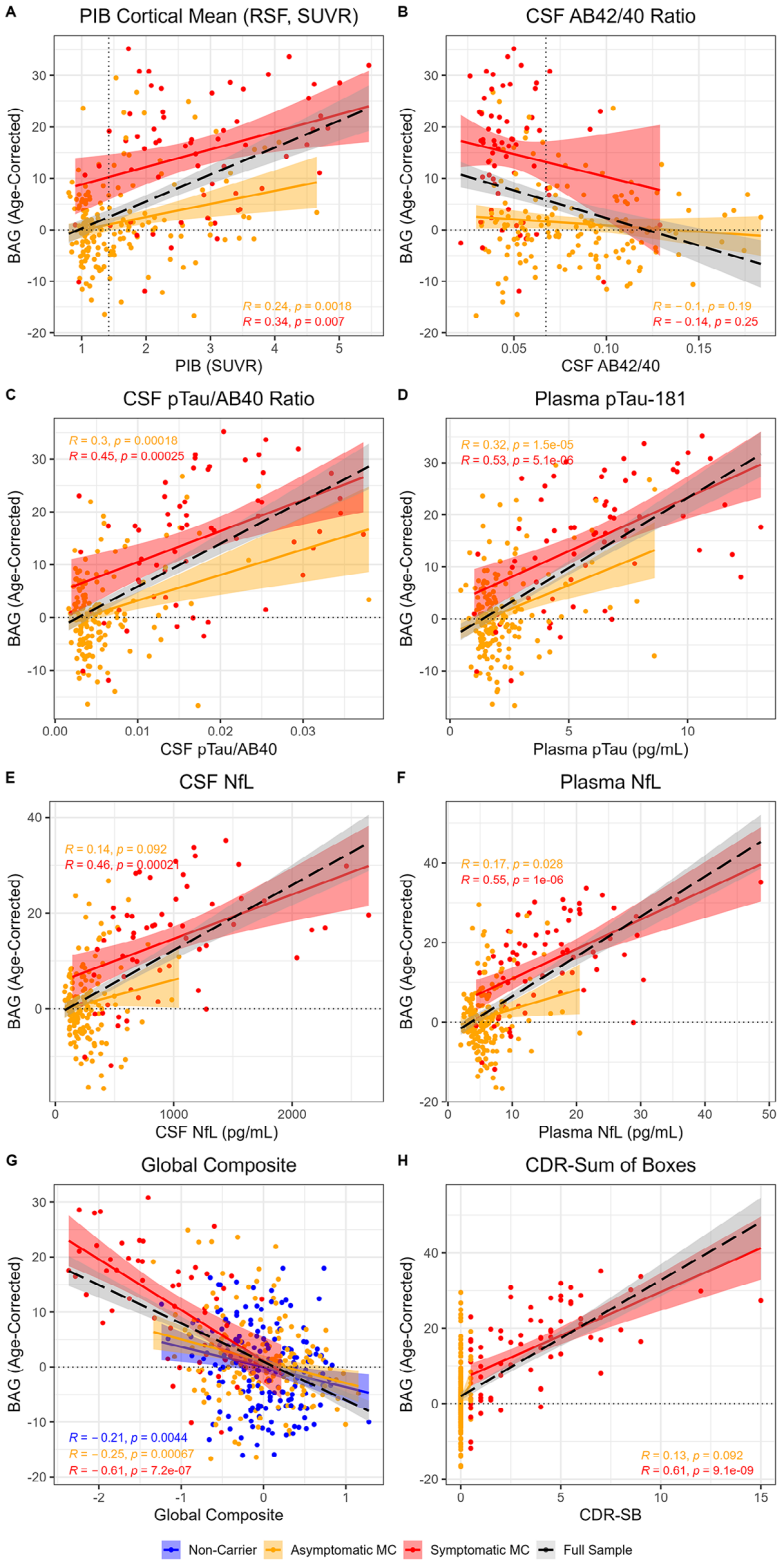
Brain age associations with amyloid biomarkers (**A **& **B**), pTau biomarkers (**C **& **D**), neurodegeneration biomarkers (**E **& **F**), and cognition (**G **& **H**). Solid colored lines and shaded regions reflect regression fits and confidence intervals derived from a linear regression model for specific groups. Dashed black lines and grey regions reflect linear regression fits in the full sample. Dotted horizontal lines reflect perfect age prediction. Dotted vertical lines reflect positivity thresholds for PIB PET and CSF Aβ42/40. Pearson’s correlation coefficient is reported for each specific group

In MCs, greater BAG was associated with greater amyloid PET uptake (PIB PET; *β* = 5.08, *p* < 0.001, *η*_*p*_^*2*^ = 0.25). As shown in Fig. 3A, amyloid PET was positively correlated with BAG in symptomatic (*r* = 0.34, *p* = 0.007) and asymptomatic MCs (*r* = 0.24, *p* = 0.002).

Greater BAG was also associated with lower CSF amyloid β 42/40 (Aβ42/40; *β* = -97.75, *p* < 0.001, *η*_*p*_^*2*^ = 0.09). However, this main effect was driven by group differences in both measures, as there were no associations between CSF Aβ42/40 and BAG within either the symptomatic (*r* = -0.14, *p* = 0.25) or the asymptomatic MCs (*r* = -0.10, *p* = 0.19; see Fig. 3B).

### Relationships with pTau markers

Greater BAG was associated with higher levels of phosphorylated tau-181 (pTau) in CSF (pTau/Aβ40 ratio; *β* = 803.71, *p* < 0.001, *η*_*p*_^*2*^ = 0.37) and plasma (*β* = 2.59, *p* < 0.001, *η*_*p*_^*2*^ = 0.37). As shown in Fig. 3C and D, measures of pTau were positively correlated with BAG in symptomatic (CSF *r* = 0.45, *p* < 0.001; plasma *r* = 0.53, *p* < 0.001) and asymptomatic MCs (CSF *r* = 0.30, *p* < 0.001; plasma *r* = 0.32, *p* < 0.001).

### Relationships with neurodegeneration markers

Greater BAG was associated with higher levels of neurofilament-light-chain (NfL) in CSF (*β* = 0.01, *p* < 0.001, *η*_*p*_^*2*^ = 0.30) and plasma (*β* = 0.97, *p* < 0.001, *η*_*p*_^*2*^ = 0.36). As shown in Fig. 3E and F, measures of NfL were positively correlated with BAG in symptomatic MCs (CSF *r* = 0.46, *p* < 0.001; plasma *r* = 0.55, *p* < 0.001). In asymptomatic MCs, BAG was positively correlated with plasma NfL (*r* = 0.17, *p* = 0.03), but showed a positive trend in relation to CSF NfL (CSF *r* = 0.14, *p* = 0.09).

There was clear evidence of skewed distributions in the amyloid, pTau, and neurodegeneration biomarker values. Thus, as a sensitivity analysis, we repeated the association analyses after log-transforming each biomarker. As shown in Supplementary Fig. 5, interpretations of the biomarker association analyses were consistent after applying the log-transformation.

### Relationships with cognition

In the full sample of MCs and NCs, greater BAG was associated with lower scores on a global cognitive composite (*β* = -6.56, *p* < 0.001, *η*_*p*_^*2*^ = 0.19). This main effect was further characterized by a group by cognition interaction (*β* = -6.16, *p* = 0.002, *η*_*p*_^*2*^ = 0.03). As shown in Fig. 3G, global cognition was most negatively correlated with BAG in symptomatic MCs (*r* = -0.61, *p* < 0.001); weaker negative correlations were also observed in asymptomatic MCs (*r* = -0.25, *p* < 0.001) and NCs (*r* = -0.21, *p* = 0.004). BAG partially mediated associations between cognition and all AD biomarkers, except a trend for plasma pTau-181 and no mediation for plasma NfL, see Supplementary Table 3 and Supplementary Fig. 6.

In MCs, greater BAG was also associated with greater cognitive impairment, as measured with the Clinical Dementia Rating® Sum-of-Boxes (CDR®-SB; *β* = 2.92, *p* < 0.001, *η*_*p*_^*2*^ = 0.38). As shown in Fig. 3H, CDR-SB was positively correlated with BAG in symptomatic MCs (*r* = 0.61, *p* < 0.001). Asymptomatic MCs are included for visualization of the full range, but were not analyzed separately, due to low variance in score.

### Comparison to other MRI measures

We examined the points of divergence between MCs and NCs in other measures derived from the same structural MRI scans as the BAG prediction. An estimate of cortical thickness in AD-specific signature regions (ICC = 0.90), precuneus thickness (ICC = 0.91), and hippocampus volume (ICC = 0.92), all achieved excellent test-retest reliability, similar to DBN-predicted brain age (ICC = 0.94) and BAG (ICC = 0.90).

**Fig. 4:**
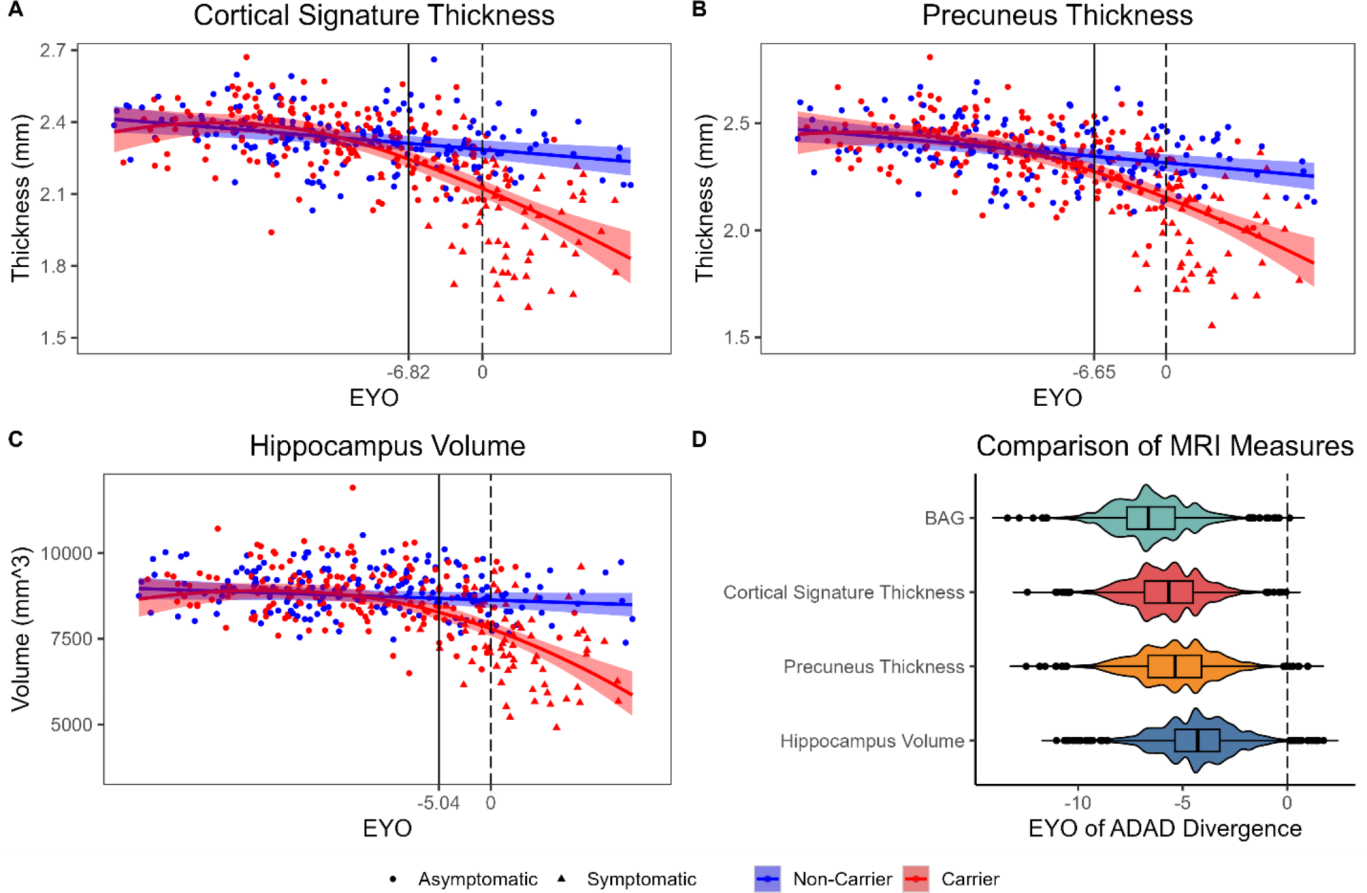
ADAD mutation differences in MRI measures over the course of disease progression. Scatterplots of cortical signature thickness (**A**), precuneus thickness (**B**), and hippocampus volume (**C**), as a function of EYO. Curves and shaded regions reflect regression fits derived from generalized additive mixed models (GAMMs) and simulation-based simultaneous 83.4% confidence intervals for comparison of two groups. Vertical dashed lines reflect EYO of 0. Vertical solid lines reflect the earliest EYOs at which MCs significantly differed from NCs. Axis labels and outlier points have been censored to prevent unblinding of participant mutation-carrier status. **D**. Violin plot of the earliest EYOs at which MCs significantly differed from NCs in the four MRI measures in 10,000 bootstrapped simulations

MCs diverged from NCs before expected symptom onset for the cortical signature (-6.82 EYO, Fig. 4A), precuneus thickness (-6.65 EYO, Fig. 4B), and hippocampus volume (-5.04 EYO, Fig. 4C). We compared the EYO of ADAD divergence between these four MRI measures with a bootstrapping analysis in 10,000 randomly resampled simulations of the full dataset. Divergence between MCs and NCs occurred significantly before EYO 0 in all MRI measures (Fig. 4D). In most simulations, the earliest EYO of divergence tended to appear in BAG (median [95% CI] = -6.64 [-9.61, -3.22]). However, this difference was not significantly different from the EYOs of divergence observed for the cortical signature (-5.67 [-8.83, -2.63], *p* = 0.36), precuneus thickness (-5.36 [-8.83, -1.83], *p* = 0.27), or hippocampus volume (-4.29 [-7.79, -1.00], *p* = 0.16).

### ADAD pathogenic variants

**Fig. 5:**
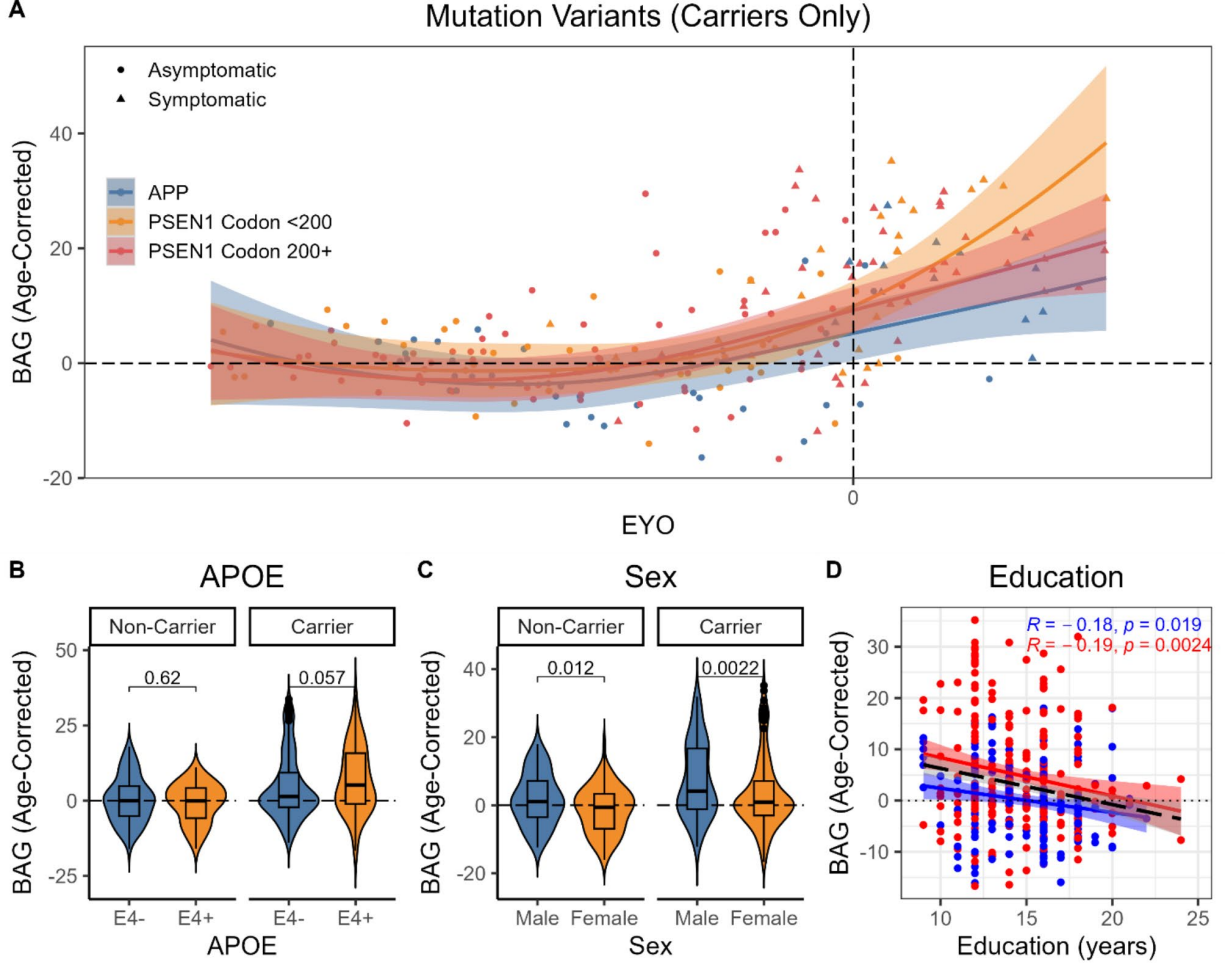
Heterogeneity in brain age gap in relation to mutation variants in MCs only (**A**), as well as *APOE* genotype (**B**), sex (**C**), and education (**D**) in the full sample

Given the low number of participants with mutations in *PSEN2*, we limited our analyses of mutation variants to carriers of mutations in *APP* or *PSEN1*, which was further split between *PSEN1* mutations before versus after codon 200. A GAMM in MCs identified a nonlinear relationship between age-corrected BAG and EYO in *APP* mutation carriers, controlling for sex, education, and *APOE*, *EDF* = 2.68, *p* < 0.001. There was a nonlinear trend, such that BAG tended to be greater in carriers of *PSEN1* mutations before codon 200 after EYO 0, *EDF* = 2.59, *p* = 0.06, but not in carriers of *PSEN1* mutations after codon 200, *EDF* = 1.00, *p* = 0.17 (see Fig. 5A).

### APOE

GAMM analyses also identified an interaction between mutation status and *APOE* ε4 positivity, *β* = 3.18, *p* = 0.008. As shown in Fig. 5B, there was a trend, such that BAG tended to be greater in ε4 + than ε4- MCs, *p* = 0.06, but did not differ between ε4 + and ε4- NCs, *p* = 0.62. *APOE* ε4 positivity did not interact with EYO in MCs, *EDF* = 1.00, *p* = 0.43, indicating that this trend was relatively consistent over the course of ADAD progression (see Supplementary Fig. 7).

### Sex differences

BAG was about 3 years greater in male than female participants, *β* = -3.15, *p* < 0.001. As shown in Fig. 5C, this sex difference was consistent in both NCs, *p* = 0.012, and MCs, *p* = 0.002. Sex did not interact with EYO in MCs, *EDF* = 1.06, *p* = 0.56, indicating that this main effect was relatively consistent over the course of ADAD progression (see Supplementary Fig. 7).

### Education

Finally, years of education were inversely associated with BAG, *β* = -0.38, *p* = 0.008. As shown in Fig. 5D, this main effect was consistent in both NCs, *r* = -0.18, *p* = 0.02, and MCs, *r* = -0.19, *p* = 0.002.

## Discussion

We applied a convolutional neural network that was previously trained to predict age from minimally-processed, whole-brain structural MRI scans to the unique DIAN cohort in order to evaluate brain age in the context of ADAD. This model accurately predicted age in the independent NCs and detected advanced brain aging in ADAD MCs, beginning almost 7 years before expected symptom onset. Brain age estimates associated with fluid-based AD biomarkers, particularly pTau and NfL, as well as cognition, and followed a similar trajectory to other MRI-based markers that require more involved manual preprocessing. Finally, brain age estimates identified heterogeneity between carriers of different ADAD mutation variants, and in relation to sex, education, and global cognition in both mutation carriers and non-carriers.

### Brain aging in ADAD

Structural brain age estimates were elevated by 13.4 years in symptomatic ADAD mutation-carriers, compared to non-carriers (Cohen’s *d* [95% CI] = 1.72 [1.41, 2.03]). The ADAD group difference is quite large, compared to prior studies of structural brain aging in sLOAD, which typically report group differences of 5–10 years using different brain age models [6, 7]. The original DeepBrainNet study also reported comparably smaller effect sizes for sLOAD (*d* = 1.26 [1.04, 1.48]) and MCI (*d* = 0.62 [0.42, 0.82]). Additionally, we also find a similar effect size to MCI when applying DeepBrainNet to an independent sLOAD sample from the Knight ADRC, including 292 cognitively unimpaired and 183 cognitively impaired older adults (*d* = 0.64 [0.45, 0.83], see Supplementary Table 4).

Differences in the magnitude of brain age elevation between AD forms may reflect differences in the spatial distribution of AD-related atrophy, which tends to be more pronounced in cortical and parietal areas in ADAD [48]. This pattern may “appear older” to brain age models than the medial temporal and limbic patterns of atrophy observed in typical sLOAD. However, prior evaluations of MRI feature importance suggest that DBN predictions of age are most strongly related to ventricular and subcortical, as opposed to cortical, regions [20, 31]. Alternatively, the stronger pattern of advanced brain age in ADAD compared to sLOAD may be a consequence of differences in the magnitude, rather than spatial pattern, of structural differences between the two AD forms. Indeed, prior studies have demonstrated that ADAD participants exhibit more pronounced cross-sectional differences in cortical thickness [48] and faster rates of longitudinal hippocampal volume loss [61], compared to sLOAD. Finally, it is also possible that differences in brain age elevation between the two AD forms might be driven by non-linear trajectories of age-related atrophy in the normative model. For instance, a comparable magnitude of AD-related atrophy might correspond to a larger deviation from the normative aging model in the younger ADAD cohort (mean age = 46.3, SD = 8.7) than it would in older sLOAD cohorts (mean ages around 75 in prior brain age studies [6, 7]).

This finding extends recent descriptions of advanced *functional connectivity-based* brain aging in ADAD, measured using resting-state fMRI measures of network organization [19]. In contrast to the network-based approach, the structural brain age model achieves better age prediction accuracy, follows a clearer trajectory over ADAD progression, and demonstrates stronger associations with AD biomarkers. These differences are consistent with observations in sLOAD, in which brain age models using structural MRI achieve greater prediction accuracy, as well as stronger associations with AD progression and biomarkers, than models using functional connectivity [15].

Most prior studies of structural brain age in sLOAD have only reported elevated brain age [6, 7] or associations between brain age and AD biomarkers [12, 13] in the symptomatic stages of AD, MCI, or subjective cognitive decline, but not in the preclinical stage [14–16]. Here we note that structural brain age estimates begin to diverge in asymptomatic ADAD MCs almost 7 years before expected symptom onset. We also note significant associations between brain age and AD biomarkers, including amyloid PET and pTau, even in asymptomatic MCs. Thus, structural brain age may be more sensitive to pathological changes in preclinical ADAD than in preclinical sLOAD. This pattern might suggest that ADAD is marked by a more severe and/or earlier stage of preclinical neurodegeneration than sLOAD. It is also possible that this effect reflects a greater level of resilience to accumulating AD neuropathology in the younger ADAD sample, who may have greater cognitive reserve [62] and are less likely to experience concurrent age-related neuropathology [17, 18]. Alternatively, this finding may highlight the advantage of staging ADAD participants using EYO over preclinical staging in sLOAD, which is typically determined by a dichotomized indication of amyloid positivity. Thus, advanced brain aging in preclinical sLOAD might be revealed with more precise staging.

BAG was strongly associated with NfL (both CSF and plasma) and only moderately associated with amyloid-β (PET, but not CSF). These observations suggest that MRI estimates of brain age, like NfL, capture a neurodegeneration-related signal in ADAD [38, 63]. However, the strong associations between BAG and pTau (in CSF and plasma) are somewhat surprising. A growing body of evidence indicates that pTau (particularly pTau-181 used here) is primarily associated with earlier-stages of amyloid pathology, rather than later tau aggregation or neurodegeneration [64, 65]. Our results suggest that the relationships between brain aging, amyloid-β, and pTau may be more complex than expected, at least in the context of ADAD.

### Comparison of MRI measures

Brain age estimates from DBN achieved excellent test-retest reliability that was comparable to other established MRI biomarkers of AD (i.e., hippocampus volume, precuneus thickness, and cortical thickness in AD signature regions). This finding is consistent with recent observations that DBN shows high reliability in other samples [66, 67], but see [68]. Further, deviations between MCs and NCs emerged at a similar stage of ADAD progression for all MRI markers (i.e., ~ 5–7 years before expected symptom onset). Thus, elevated structural brain age is likely driven by ADAD-related neurodegeneration in the presymptomatic and early symptomatic stages of the disease, similar to other MRI-based measures.

Structural brain age estimates (particularly from DBN) possess additional unique qualities that convey competitive advantage compared to other MRI measures. First, DBN can be estimated quickly and automatically using a publicly-available Python script, which requires less intensive preprocessing and quality control [20]. In contrast, the other MRI biomarkers tested in this study require multiple stages of preprocessing and manual quality control in FreeSurfer [18, 47, 48], potentially limiting practical implementation. Second, BAG estimates offer a unique interpretability in terms of “years” or “brain age”, which may be more accessible to patients or lay public than typical MRI measurement units of regional volume or thickness. Third, brain age predictions were also associated with cognitive performance, years of education, and sex differences in non-carriers (see below for further discussion). Indeed, other studies have also demonstrated that brain age estimates are sensitive to a wide range of neurological, psychiatric, and general health conditions [6, 7]. Thus, structural brain age might be useful more broadly as a general screening measure of brain health, disease risk, or resilience, compared to other MRI features, which may be more specific to AD-related pathology.

### Heterogeneity in brain aging

We found that elevated structural brain age was not consistently observed in all ADAD mutation variants. Elevated brain age was most strongly observed in participants carrying a *PSEN1* mutation before codon 200, followed by *PSEN1* mutation after codon 200, then *APP* mutation-carriers. Although this result is limited by relatively small sample sizes, it is consistent with prior demonstrations of heterogeneity in clinical presentation between ADAD pathogenic variants. While carriers of *PSEN1* mutations after codon 200 exhibit greater cerebral amyloid angiopathy [21] and white matter hyperintensities [22], carriers of mutations before codon 200 exhibit a younger age of onset [23] and greater amyloid burden [24]. Future studies should examine heterogeneity between ADAD mutation variants in larger samples to understand how differing pathological presentations influence cognitive and functional outcomes.

We also found that structural brain age estimates were significantly younger in female than male participants, including both ADAD mutation carriers and non-carriers. This finding is consistent with prior observations of sex differences in brain age estimates using metabolic PET [26, 27], structural MRI [14, 25, 29], and localized to prefrontal regions [28]. This result may also be informative on potential sex differences in resilience to AD pathology. Female participants have been shown to outperform males in cognitive tests, despite equivalent levels of AD pathology in sLOAD [69–71], Down syndrome [72], and ADAD [73], including the same DIAN cohort [74]. Our finding of younger-appearing brain age in an overlapping sample of DIAN participants suggests that this measure might capture sex differences in brain reserve [62]. These sex differences are likely multifactorial, potentially driven by interactive hormonal, genetic, and/or environmental influences [75], and may emerge earlier in development and remain through adulthood [26]. However, the main effect of sex is also somewhat surprising, considering a lack of sex differences in the original report on the development of the DBN model [20], as well as in a recent application of DBN in samples of healthy aging and sLOAD participants [66].

Lower estimates of structural brain age were associated with greater educational duration in both NCs and MCs. The interpretation of years of education in the DIAN cohort is complicated by a lack of international standardization for quantification. Yet this finding is consistent with prior observations that reduced brain age is associated with greater educational duration [30] and achievement [31] in US samples, granted these effects were noted to be small and did not survive correction for multiple comparisons [31]. If years of education is considered as a proxy of socioeconomic exposures, this result also supports the proposal that brain age is sensitive to other lifetime exposures and influences, including childhood IQ [76] and birth weight [77]. Notably, the present results also suggest that this association is consistent in ADAD, as well as control samples. Lower brain age estimates were also associated with better cognitive performance in NCs. Some prior studies have also reported associations between brain age and cognitive measures in healthy adult samples [14, 76, 78–80], but like education, these effects are small and not consistently observed [15]. Together, these findings suggest that brain age likely captures aspects of resilience or brain health more broadly, albeit with a small effect size, in addition to pathological signal.

Prior studies have demonstrated *APOE*-related differences in longitudinal, but not cross-sectional, brain age estimates [32]. Nor did *APOE* moderate brain age estimates in a prior study of Down syndrome [81], another genetic form of AD. In the present results, we observed an interactive effect, such that ε4 positivity trended towards greater brain age in ADAD mutation carriers, but not in non-carriers. Taken together, these findings suggest that the effects of *APOE* genotype on brain age estimates are relatively small and inconsistently observed.

### Limitations & future directions

Structural MRI scans from DIAN were collected over multiple international sites including a range of scanner models, which may introduce confounding noise into MRI features. We attempted to limit the influence of non-biological sources of variance by processing all MRI data through a common pipeline and quality assessment procedures, as well as statistically harmonizing brain age predictions across sites and scanners with ComBat [53]. Additionally, the sample size for analyses with other AD biomarkers differed across each measure, and these analyses did not include more recent measures of tau, including tau PET, microtubule-binding regions, or additional tau phosphorylation sites. Future analyses should replicate these results in more complete biomarker samples to more thoroughly evaluate associations with established AD biomarkers. Finally, the DIAN sample is mostly non-Hispanic white and highly educated. Future studies would benefit from models that are trained and tested on more diverse samples to ensure broad generalizability.

## Conclusions

These results present clear evidence of advanced structural brain aging in the late presymptomatic and early symptomatic stages of ADAD, likely reflecting neurodegenerative processes. Although atrophy can indeed be detected using other MRI indicators, brain age estimates offer comparative advantages including ease of processing and simplicity of interpretation, while maintaining comparable reliability and sensitivity to ADAD. Brain age estimates are likely also sensitive to additional sources of disease risk, resilience, and general health, including global cognition, education, and sex differences. These results build upon prior evidence suggesting that brain age models may offer potential utility as a general screening measure of brain health.

## Appendix 1

Dominantly Inherited Alzheimer Network consortium members:

James M. Noble^1^; Gregory S. Day^2^; Neill R. Graff-Radford^2^; Jonathan Vöglein^3^; Johannes Levin^3^; Ricardo F. Allegri^4^; Patricio Chrem Mendez^4^; Ezequiel Surace^4^; Sarah B. Berman^5^; Snezana Ikonomovic^5^; Neelesh K. Nadkarni^5^; Francisco Lopera^6^; Laura Ramirez^6^; David Aguillon^6^; Yudy Leon^6^; Claudia Ramos^6^; Diana Alzate^6^; Ana Baena^6^; Natalia Londono^6^; Sonia Moreno^6^; Mathias Jucker^7^; Christoph Laske^7^; Elke Kuder-Buletta^7^; Susanne Graber-Sultan^7^; Oliver Preische^7^; Anna Hofmann^7^; Takeshi Ikeuchi^8^; Kensaku Kasuga^8^; Yoshiki Niimi^9^; Kenji Ishii^8,9^; Michio Senda^10^; Raquel Sanchez-Valle^11^; Pedro Rosa-Neto^12^; Nick C. Fox^13^; Dave Cash^13^; Jae-Hong Lee^14^; Jee Hoon Roh^15^; Meghan C. Riddle^16^; William Menard^16^; Courtney Bodge^16^; Mustafa Surti^16^; Leonel Tadao Takada^17^; Martin Farlow^18^; Jasmeer P. Chhatwal^19^; VJ Sanchez-Gonzalez^20^; Maribel Orozco-Barajas^20^; Alison M. Goate^21^; Alan E. Renton^21^; Bianca T. Esposito^21^; Celeste M. Karch^22^; Jacob Marsh^22^; Carlos Cruchaga^22^; Victoria Fernanadez^22^; Brian A. Gordon^22^; Anne M. Fagan^22^; Gina Jerome^22^; Elizabeth Herries^22^; Jorge Llibre-Guerra^22^; Allan I. Levey^23^; Erik C.B. Johnson^23^; Nicholas T. Seyfried^23^; Peter R. Schofield^24^; William S. Brooks^24^; Jacob A Bechara^24^; Randall Bateman^22^***; Eric McDade^22^; Jason Hassenstab^22^; Richard J. Perrin^22^; Erin E. Franklin^22^; Tammie Benzinger^22^; Allison Chen^22^; Charles Chen^22^; Shaney Flores^22^; Nelly Friedrichsen^22^; Brian Gordon^22^; Nancy Hantler^22^; Russ Hornbeck^22^; Steve Jarman^22^; Sarah Keefe^22^; Deborah Koudelis^22^; Parinaz Massoumzadeh^22^; Austin McCullough^22^; Nicole McKay^22^; Joyce Nicklaus^22^; Christine Pulizos^22^; Qing Wang^22^; Sheetal Mishall^22^; Edita Sabaredzovic^22^; Emily Deng^22^; Madison Candela^22^; Hunter Smith^22^; Diana Hobbs^22^; Jalen Scott^22^; Chengjie Xiong^22^; Peter Wang^22^; Xiong Xu^22^; Yan Li^22^; Emily Gremminger^22^; Yinjiao Ma^22^; Ryan Bui^22^; Ruijin Lu^22^; Ralph Martins^25^; Ana Luisa Sosa Ortiz^26^; Alisha Daniels^22^; Laura Courtney^22^; Hiroshi Mori^10^; Charlene Supnet-Bell^22^; Jinbin Xu^22^; John Ringman^27^; Nicolas Barthelemy^22^; John Morris^22^; Jennifer Smith^22^.

1 Columbia University, New York City, USA.

2 Mayo Clinic, Jacksonville, USA.

3 German Center for Neurodegnerative Diseases (DZNE), Munich, Germany.

4 Institute of Neurological Research Fleni, Buenos Aires, Argentina.

5 University of Pittsburgh, Pittsburgh, USA.

6 Universidad de Antioquia, Medellín, Colombia.

7 German Center for Neurodegenerative Diseases (DZNE), Tübingen, Germany.

8 Niigata University, Niigata, Japan.

9 University of Tokyo, Tokyo, Japan.

10 Osaka Metropolitan University, Osaka, Japan.

11 Clínic de Barcelona, Barcelona, Spain.

12 McGill University, Montreal, Canada.

13 University College London, London, United Kingdom.

14 Asan Medical Center, Seoul, Republic of Korea.

15 Korea University College of Medicine, Seoul, Republic of Korea.

16 Brown University, Providence, USA.

17 University of São Paulo, São Paulo, Brazil.

18 Indiana University, Indianapolis, USA.

19 Massachusetts General Hospital, Boston, USA.

20 Universidad de Guadalajara, Jalisco, México.

21 Icahn School of Medicine at Mt. Sinai, New York City, USA.

22 Washington University in St. Louis, St. Louis, USA.

23 Emory University, Atlanta, USA.

24 Neuroscience Research Australia, Sydney, Australia.

25 Edith Cowan University, Perth, Australia.

26 Instituto Nacional de Neurologia y Neurocirugía MVS, Ciudad de México, Mexico.

27 University of Southern California, Los Angeles, USA.

*** Consortium representative: batemanr@wustl.edu.

### Electronic supplementary material

Below is the link to the electronic supplementary material.

Supplementary Material 1

## Data Availability

This study utilized datasets obtained from the DIAN Observational Study (Data Freeze 16). The data are available to all qualified researchers after appropriate review. Requests for data access may be placed to the DIAN Steering Committee (https://dian.wustl.edu/our-research/for-investigators/dian-observational-study-investigator-resources/data-request-terms-and-instructions/). Code used in this study is available at https://github.com/peterrmillar/DIANBrainAge.

## List of abbreviations

AD: Alzheimer disease
ADAD: autosomal dominant AD
APOE: apolipoprotein E
APP: amyloid precursor protein
Aβ40: amyloid β42
Aβ42: amyloid β42
BAG: brain age gap
CDR®: Clinical Dementia Rating®
CSF: cerebrospinal fluid
DBN: DeepBrainNet
DIAN: Dominantly Inherited Alzheimer Network
EYO: estimated years until symptom onset
FDR: false discovery rate
FOV: field of view
GAMM: generalized additive mixed model
ICC: intraclass correlation coefficient
LME: linear mixed effects
MAE: mean absolute error
MC: mutation-carrier
MPRAGE: magnetization-prepared rapid gradient echo
MRI: magnetic resonance imaging
NC: non-carrier
NfL: neurofilament-light-chain
PET: positron emission tomography
PiB: Pittsburgh Compound B
PSEN1: presenilin-1
PSEN2: presenilin-2
pTau: phosphorylated tau-181
PUP: PET Unified Pipeline
RMSE: root mean square error
RSF: regional spread function
sLOAD: sporadic late onset AD
SUVR: standardized uptake value ratio
TE: echo time
TI: inversion time
TR: repetition time
