# Supplementary material for: Advanced structural brain aging in preclinical autosomal dominant Alzheimer disease
Source: Mol Neurodegener. 2023 Dec 19;18:98. doi: 10.1186/s13024-023-00688-3 (PMC10729487; doi:10.1186/s13024-023-00688-3)
Supplement: Supplementary file 1 — Supplementary Material 1 [file 13024_2023_688_MOESM1_ESM.docx]

**Supplementary Material**

**Supplementary Figure 1.** Association between BAG and chronological age before (**A**) and after (**B**) applying a linear correction to predicted brain age values. A non-significant negative association between BAG and age is observed in NCs, consistent with the common regression dilution effect, however it is not observed in MCs. The bias in NCs is removed after linear correction. Solid colored lines and shaded regions reflect regression fits and confidence intervals derived from a linear regression model for specific groups. Dashed black lines and grey regions reflect linear regression fits in the full sample. Dotted horizontal lines reflect a perfect match between predicted and chronological age. Pearson’s correlation coefficient is reported for each specific group.


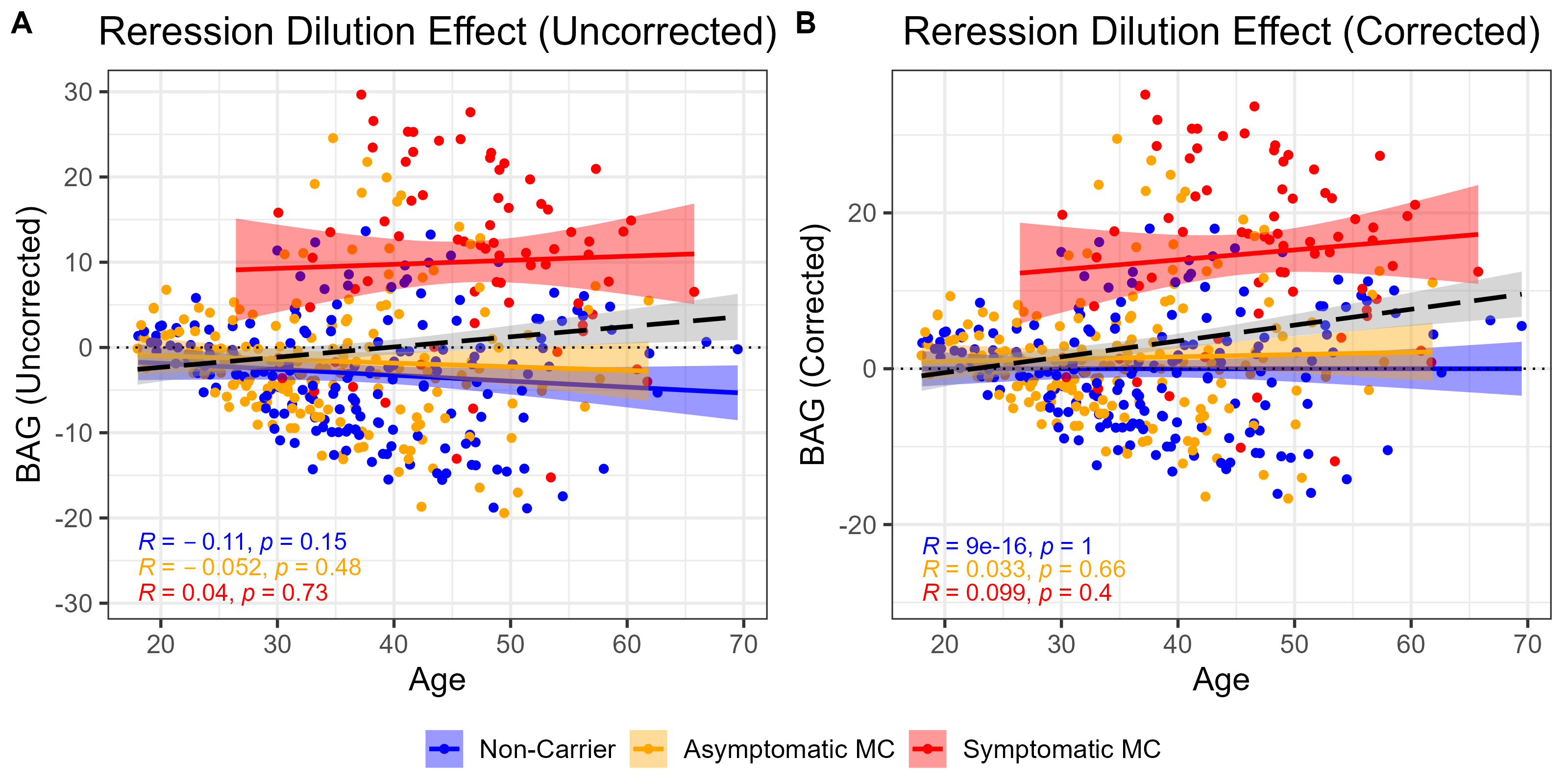


**Supplementary Table 1 (next page).** Demographic information for each site. CDR = Clinical Dementia Rating. EYO = Estimated years until symptom onset. *APOE* = Apolipoprotein E. *APP* = Amyloid Precursor Protein. *PSEN* = Presenilin.

|  | **Sites** | | | | | | | | | | | | | | | **η²** | **p-value** |
| --- | --- | --- | --- | --- | --- | --- | --- | --- | --- | --- | --- | --- | --- | --- | --- | --- | --- |
|  | **A** | **C** | **D** | **E** | **F** | **G** | **I** | **J** | **K** | **L** | **M** | **N** | **O** | **P** | **Q** |  |  |
| **N** | 102 | 23 | 33 | 8 | 34 | 29 | 5 | 23 | 30 | 33 | 38 | 33 | 31 | 9 | 5 |  |  |
| **Age** | 36.9 (10.7) | 35.3 (11.1) | 31.8 (9.8) | 48.6 (7.3) | 38.3 (9.7) | 40.5 (11.2) | 38.4 (2.0) | 41.5 (11.8) | 37.2 (9.2) | 37.8 (9.6) | 38.2 (9.4) | 39.2 (11.5) | 37.9 (11.6) | 36.2 (9.0) | 33.7 (4.6) | 0.063 | 0.013 |
| **Sex** |  |  |  |  |  |  |  |  |  |  |  |  |  |  |  |  | 0.062 |
| *Female* | 49 (48%) | 16 (70%) | 25 (76%) | 4 (50%) | 22 (65%) | 16 (55%) | 2 (40%) | 15 (65%) | 16 (53%) | 23 (70%) | 20 (53%) | 13 (39%) | 14 (45%) | 6 (67%) | 1 (20%) |  |  |
| *Male* | 53 (52%) | 7 (30%) | 8 (24%) | 4 (50%) | 12 (35%) | 13 (45%) | 3 (60%) | 8 (35%) | 14 (47%) | 10 (30%) | 18 (47%) | 20 (61%) | 17 (55%) | 3 (33%) | 4 (80%) |  |  |
| **Education** | 15.4 (2.6) | 14.5 (2.5) | 14.8 (2.4) | 14.5 (2.3) | 15.1 (3.1) | 15.1 (2.3) | 13.8 (3.5) | 14.3 (2.1) | 13.4 (2.7) | 13.8 (2.6) | 13.6 (2.5) | 14.6 (3.0) | 16.0 (4.7) | 14.4 (2.6) | 15.2 (1.8) | 0.070 | 0.024 |
| **Mutation** |  |  |  |  |  |  |  |  |  |  |  |  |  |  |  |  | 0.024 |
| *Non-Carrier* | 52 (51%) | 9 (39%) | 8 (24%) | 1 (12%) | 14 (41%) | 16 (55%) | 2 (40%) | 8 (35%) | 9 (30%) | 12 (36%) | 12 (32%) | 10 (30%) | 20 (65%) | 3 (33%) | 3 (60%) |  |  |
| *Carrier* | 50 (49%) | 14 (61%) | 25 (76%) | 7 (88%) | 20 (59%) | 13 (45%) | 3 (60%) | 15 (65%) | 21 (70%) | 21 (64%) | 26 (68%) | 23 (70%) | 11 (35%) | 6 (67%) | 2 (40%) |  |  |
| **CDR** |  |  |  |  |  |  |  |  |  |  |  |  |  |  |  |  | 0.093 |
| *0* | 92 (90%) | 21 (91%) | 26 (79%) | 7 (88%) | 24 (71%) | 23 (79%) | 3 (60%) | 18 (78%) | 23 (77%) | 31 (94%) | 29 (76%) | 25 (76%) | 28 (90%) | 7 (78%) | 5 (100%) |  |  |
| *0.5* | 9 (8.8%) | 2 (8.7%) | 4 (12%) | 1 (12%) | 4 (12%) | 2 (6.9%) | 2 (40%) | 3 (13%) | 3 (10%) | 1 (3.0%) | 5 (13%) | 4 (12%) | 2 (6.5%) | 2 (22%) | 0 (0%) |  |  |
| *1* | 1 (1.0%) | 0 (0%) | 2 (6.1%) | 0 (0%) | 5 (15%) | 4 (14%) | 0 (0%) | 1 (4.3%) | 4 (13%) | 1 (3.0%) | 3 (7.9%) | 4 (12%) | 1 (3.2%) | 0 (0%) | 0 (0%) |  |  |
| *2* | 0 (0%) | 0 (0%) | 1 (3.0%) | 0 (0%) | 1 (2.9%) | 0 (0%) | 0 (0%) | 1 (4.3%) | 0 (0%) | 0 (0%) | 1 (2.6%) | 0 (0%) | 0 (0%) | 0 (0%) | 0 (0%) |  |  |
| **EYO** | -13.4 (11.7) | -15.0 (9.8) | -12.2 (10.0) | -8.6 (7.3) | -8.2 (13.3) | -7.3 (11.6) | -2.6 (4.3) | -9.9 (10.9) | -6.6 (9.7) | -10.0 (10.1) | -7.5 (10.5) | -9.5 (9.9) | -10.9 (12.9) | -6.5 (5.7) | -8.5 (2.8) | 0.061 | 0.024 |
| **APOE** |  |  |  |  |  |  |  |  |  |  |  |  |  |  |  |  | 0.062 |
| *ε4-* | 66 (65%) | 12 (52%) | 24 (73%) | 3 (38%) | 23 (68%) | 21 (72%) | 5 (100%) | 15 (65%) | 18 (60%) | 25 (76%) | 27 (71%) | 29 (88%) | 19 (61%) | 9 (100%) | 4 (80%) |  |  |
| *ε4+* | 36 (35%) | 11 (48%) | 9 (27%) | 5 (62%) | 11 (32%) | 8 (28%) | 0 (0%) | 8 (35%) | 12 (40%) | 8 (24%) | 11 (29%) | 4 (12%) | 12 (39%) | 0 (0%) | 1 (20%) |  |  |
| **Variant** |  |  |  |  |  |  |  |  |  |  |  |  |  |  |  |  | <0.001 |
| *APP* | 7 (6.9%) | 4 (17%) | 3 (9.1%) | 0 (0%) | 4 (12%) | 16 (55%) | 0 (0%) | 0 (0%) | 9 (30%) | 20 (61%) | 5 (13%) | 15 (45%) | 6 (19%) | 0 (0%) | 0 (0%) |  |  |
| *PSEN1 Codon <200* | 28 (27%) | 1 (4.3%) | 6 (18%) | 2 (25%) | 14 (41%) | 9 (31%) | 0 (0%) | 1 (4.3%) | 0 (0%) | 8 (24%) | 18 (47%) | 12 (36%) | 18 (58%) | 9 (100%) | 0 (0%) |  |  |
| *PSEN1 Codon 200+* | 30 (29%) | 18 (78%) | 24 (73%) | 6 (75%) | 14 (41%) | 4 (14%) | 5 (100%) | 22 (96%) | 21 (70%) | 3 (9.1%) | 15 (39%) | 4 (12%) | 6 (19%) | 0 (0%) | 5 (100%) |  |  |
| *PSEN2* | 37 (36%) | 0 (0%) | 0 (0%) | 0 (0%) | 2 (5.9%) | 0 (0%) | 0 (0%) | 0 (0%) | 0 (0%) | 2 (6.1%) | 0 (0%) | 2 (6.1%) | 1 (3.2%) | 0 (0%) | 0 (0%) |  |  |

**
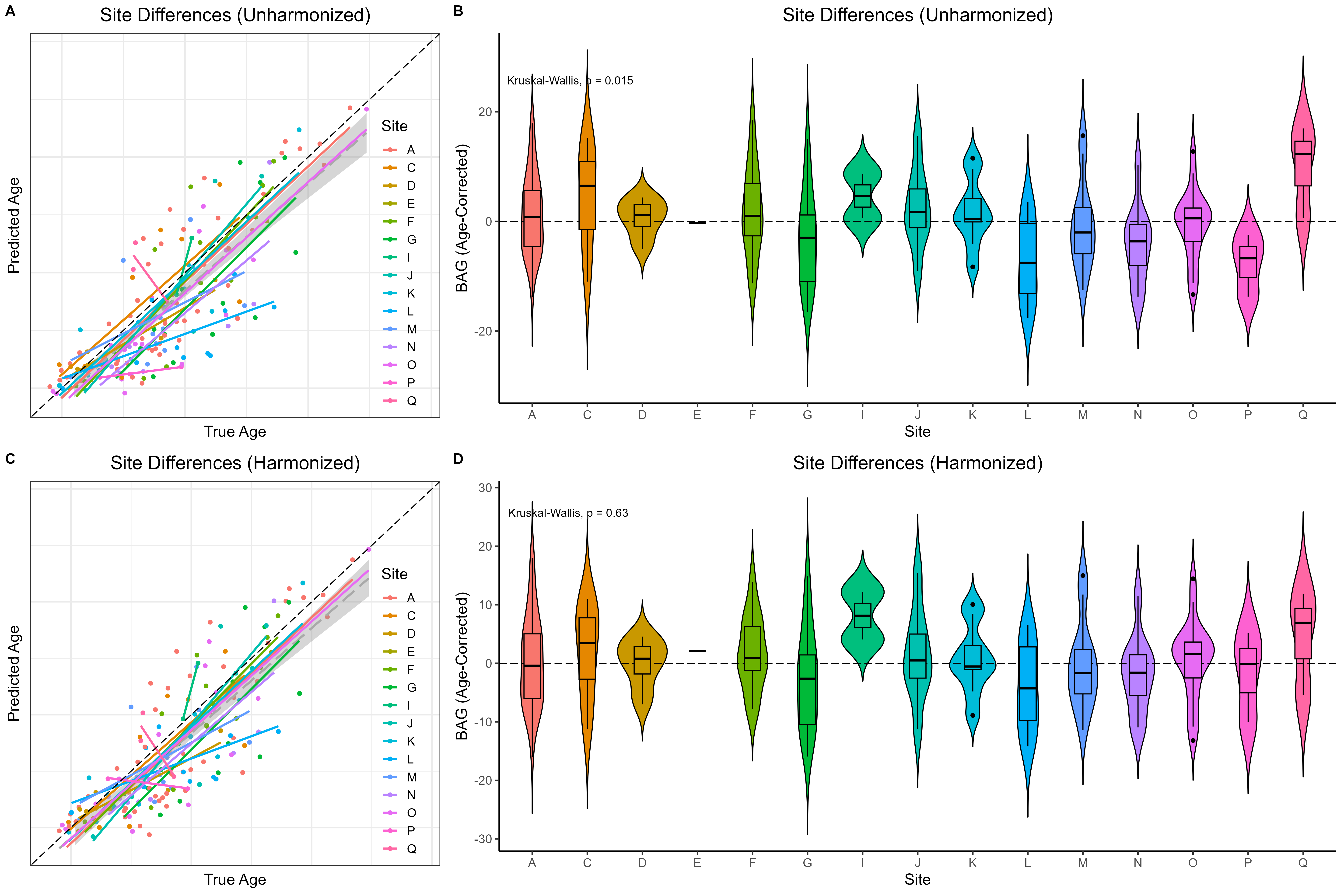
Supplementary Figure 2.** Brain age estimates in different sites before (**A**, **B**) and after harmonization (**C**, **D**). Scatterplots show brain-predicted age as a function of true age. Lines reflect regression fits. Dashed line reflects perfect age prediction. Violin plots show brain age gap (BAG) after correcting for true age. Dashed line represents a BAG of 0. *P* value is reported from a Kruskall-Wallis test of differences in BAG between sites.

**
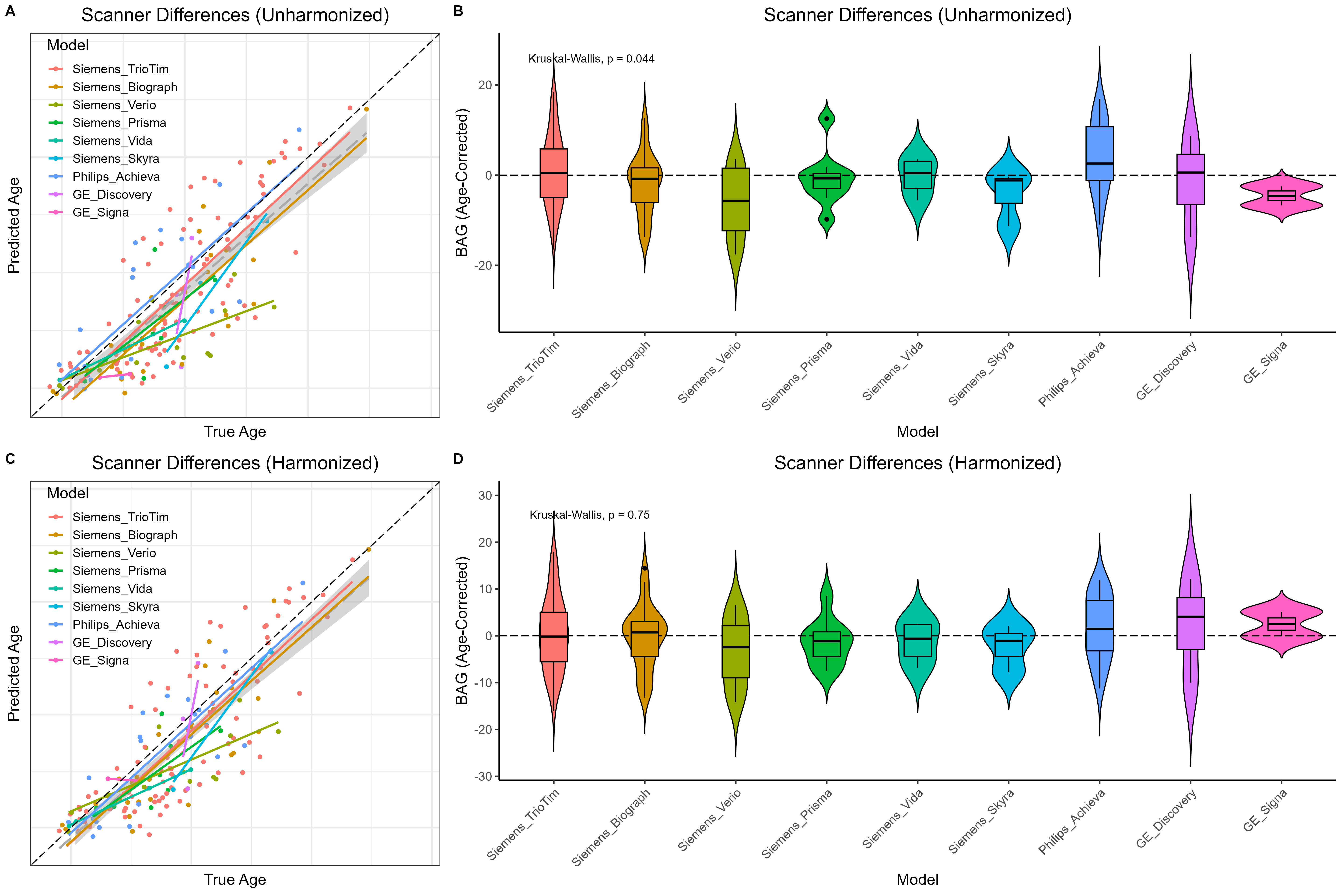
 Supplementary Figure 3.** Brain age estimates in different scanners before (**A**, **B**) and after harmonization (**C**, **D**). Scatterplots show brain-predicted age as a function of true age. Lines reflect regression fits. Dashed line reflects perfect age prediction. Violin plots show brain age gap (BAG) after correcting for true age. Dashed line represents a BAG of 0. *P* value is reported from a Kruskall-Wallis test of differences in BAG between scanners.

|  |  | **ADAD Mutation Groups** | | | **NCs vs. All MCs** | |
| --- | --- | --- | --- | --- | --- | --- |
|  | ***N*** | **NCs  (72)** | **Asymptomatic MCs  (67)** | **Symptomatic MCs  (43)** | ***η²*** | ***p*-value^1^** |
| **Time Interval (years)** | 182 |  |  |  | 0.079 | <0.001 |
| *Mean (SD)* |  | 2.53 (1.20) | 2.27 (1.24) | 1.16 (0.47) |  |  |
| **Age** | 182 |  |  |  | 0.002 | 0.57 |
| *Mean (SD)* |  | 40.1 (10.3) | 36.9 (9.5) | 47.3 (8.5) |  |  |
| **Sex** | 182 |  |  |  |  | 0.48 |
| *Female* |  | 45 (62%) | 41 (61%) | 22 (51%) |  |  |
| *Male* |  | 27 (38%) | 26 (39%) | 21 (49%) |  |  |
| **Education** | 182 |  |  |  | 0.009 | 0.20 |
| *Mean (SD)* |  | 14.9 (2.5) | 14.8 (2.8) | 13.7 (2.2) |  |  |
| **CDR** | 182 |  |  |  |  | <0.001 |
| *0* |  | 72 (100%) | 67 (100%) | 0 (0%) |  |  |
| *0.5* |  | 0 (0%) | 0 (0%) | 24 (56%) |  |  |
| *1* |  | 0 (0%) | 0 (0%) | 13 (30%) |  |  |
| *2* |  | 0 (0%) | 0 (0%) | 6 (14%) |  |  |
| **EYO** | 182 |  |  |  | 0.011 | 0.16 |
| *Mean (SD)* |  | -7.3 (10.4) | -10.4 (8.7) | 3.0 (4.5) |  |  |
| ***APOE*** | 182 |  |  |  |  | 0.51 |
| *ε4-* |  | 53 (74%) | 50 (75%) | 26 (60%) |  |  |
| *ε4+* |  | 19 (26%) | 17 (25%) | 17 (40%) |  |  |
| **Variant** | 182 |  |  |  |  | >0.99 |
| *APP* |  | 23 (32%) | 20 (30%) | 6 (14%) |  |  |
| *PSEN1 Codon <200* |  | 16 (22%) | 19 (28%) | 16 (37%) |  |  |
| *PSEN1 Codon 200+* |  | 25 (35%) | 26 (39%) | 20 (47%) |  |  |
| *PSEN2* |  | 8 (11%) | 2 (3.0%) | 1 (2.3%) |  |  |
|  | | | | | | |
| ^1^ Wilcoxon rank sum test; Pearson's Chi-squared test; Fisher's exact test | | | | | | |

**Supplementary Table 2.** Baseline demographic information for the longitudinal subset. ADAD = Autosomal Dominant Alzheimer Disease. NC = Non-carrier. MC = Mutation-carrier. CDR = Clinical Dementia Rating. EYO = Estimated years until symptom onset. *APOE* = Apolipoprotein E. *APP* = Amyloid Precursor Protein. *PSEN* = Presenilin.

**
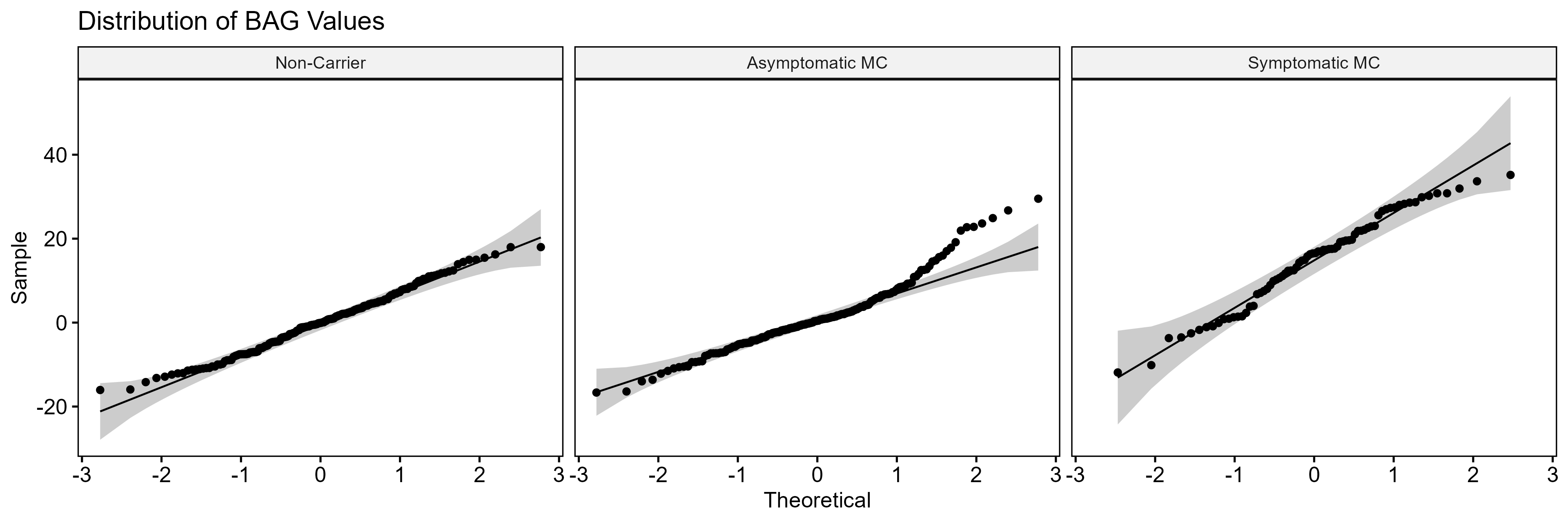
**

**Supplementary Figure 4.** Quantile-quantile plots of age-corrected brain age gap in each of the analysis sets (non-carriers, asymptomatic mutation carriers [MC], and symptomatic MC).

**Supplementary Figure 5 (next page).** Brain age associations with log-transformed amyloid biomarkers (**A** & **B**), log-transformed pTau biomarkers (**C** & **D**), and log-transformed neurodegeneration biomarkers (**E** & **F**). Solid colored lines and shaded regions reflect regression fits and confidence intervals derived from a linear regression model for specific groups. Dashed black lines and grey regions reflect linear regression fits in the full sample. Dotted horizontal lines reflect perfect age prediction. Pearson’s correlation coefficient is reported for each specific group.


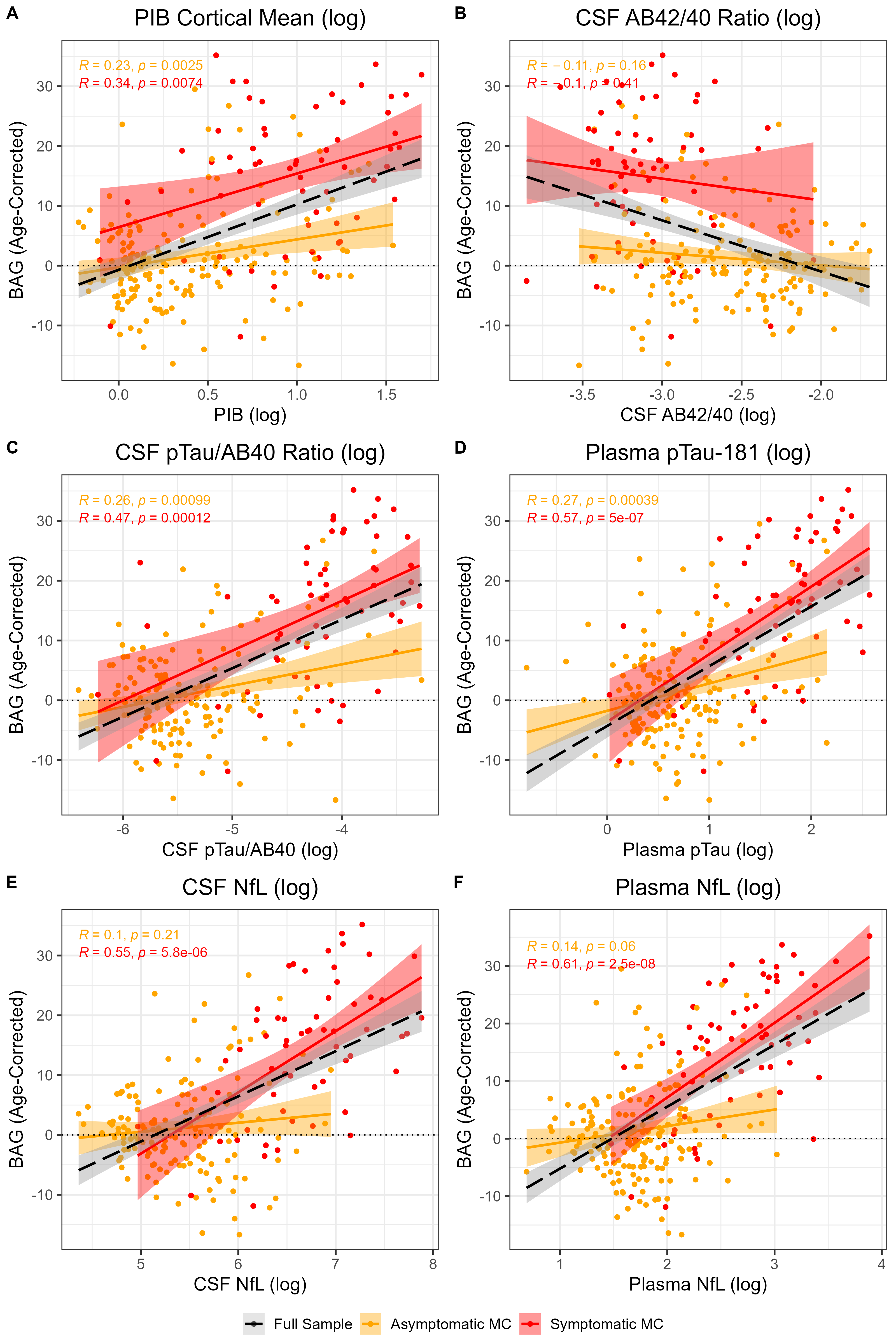


|  | **% Mediated** | **95% CI Low** | **95% CI High** | **p value** |
| --- | --- | --- | --- | --- |
| *PIB PET* | 0.53 | 0.22 | 0.94 | <2e-16 |
| *CSF AB42/40* | 0.36 | 0.22 | 0.52 | <2e-16 |
| *CSF pTau-181/AB40* | 0.55 | 0.36 | 0.92 | <2e-16 |
| *Plasma pTau-181* | 0.28 | 0.00 | 0.51 | 0.052 |
| *CSF NfL* | 0.27 | 0.14 | 0.40 | <2e-16 |
| *Plasma NfL* | 0.18 | -0.05 | 0.39 | 0.12 |

**Supplementary Table 3.** Proportional mediation by BAG of relationships between AD biomarkers and global cognitive composite.

**Supplementary Figure 6 (next page).** Results of mediation analyses testing BAG as a mediator in the relationship between AD biomarkers and global cognition in ADAD mutation carriers. ACME = Average Causal Mediation Effects. ADE = Average Direct Effects. Horizontal lines reflect 95% confidence intervals derived from a non-parametric bootstrap method with 1000 simulations [60].


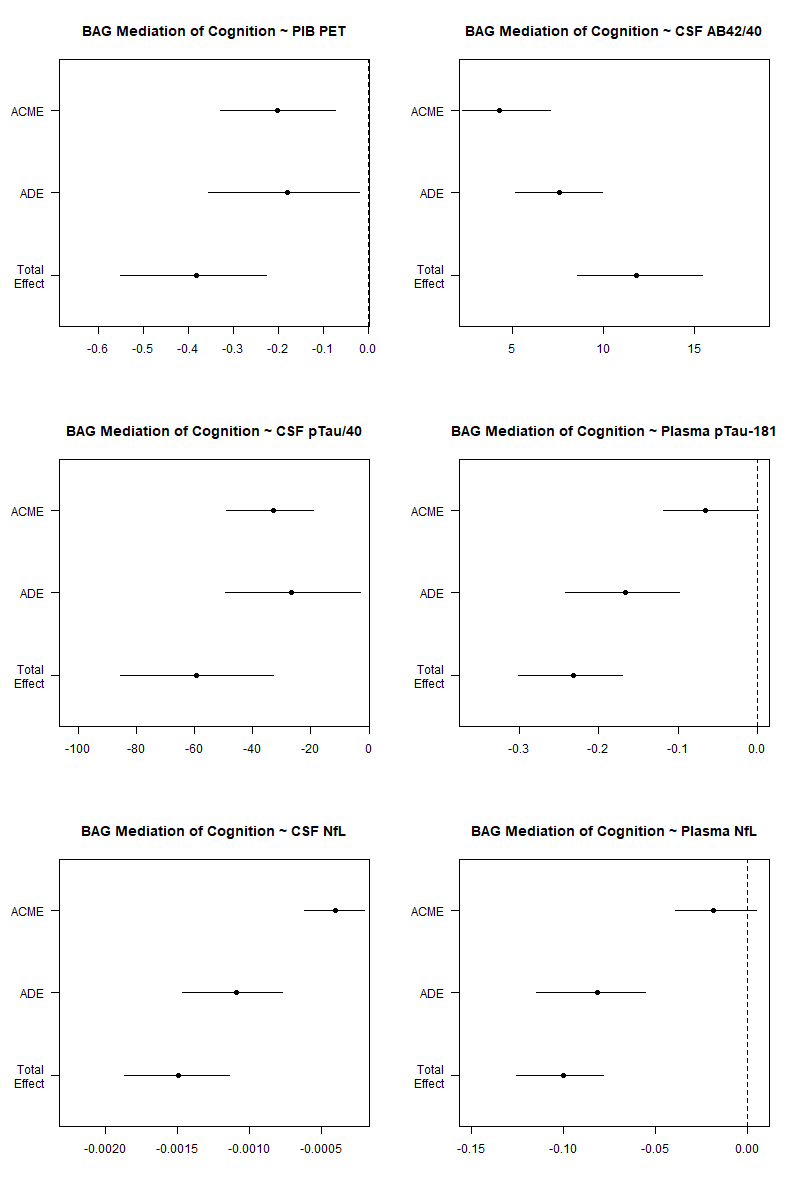


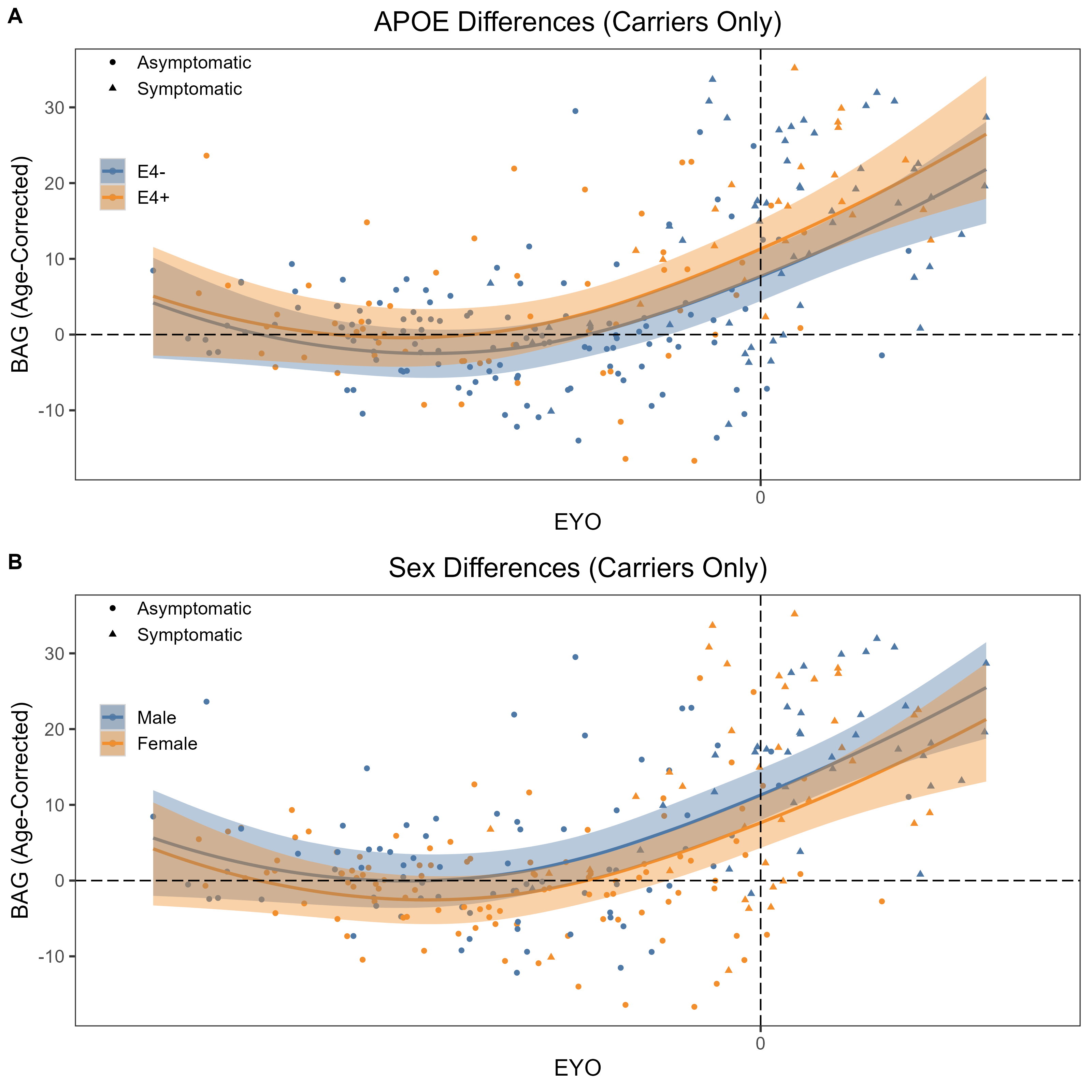


**Supplementary Figure 7.** Heterogeneity in brain age gap in relation to *APOE* genotype (**A**) and sex (**B**) in MCs only.

|  |  | **sLOAD Groups** | | | |
| --- | --- | --- | --- | --- | --- |
|  | ***N*** | **CU  (292)** | **CI  (183)** | ***η²*** | ***p*-value^1^** |
| **Age** | 475 |  |  | 0.105 | <0.001 |
| *Mean (SD)* |  | 69.9 (8.3) | 75.4 (6.9) |  |  |
| **Sex** | 475 |  |  |  | <0.001 |
| *Female* |  | 114 (39%) | 106 (58%) |  |  |
| *Male* |  | 178 (61%) | 77 (42%) |  |  |
| **Education** | 475 |  |  | 0.017 | 0.006 |
| *Mean (SD)* |  | 15.8 (2.7) | 15.1 (3.0) |  |  |
| **CDR** | 475 |  |  |  | <0.001 |
| *0* |  | 292 (100%) | 0 (0%) |  |  |
| *0.5* |  | 0 (0%) | 146 (80%) |  |  |
| *1* |  | 0 (0%) | 35 (19%) |  |  |
| *2* |  | 0 (0%) | 2 (1.1%) |  |  |
| ***APOE*** | 473 |  |  |  | <0.001 |
| *ε4-* |  | 180 (62%) | 76 (42%) |  |  |
| *ε4+* |  | 112 (38%) | 105 (58%) |  |  |
| ***BAG (Age-Corrected)*** | 475 |  |  | 0.088 | <0.001 |
| *Mean (SD)* |  | -1.2 (5.4) | 2.1 (4.8) |  |  |

**Supplementary Table 4.** Demographic information for the sLOAD comparison sample from the Knight ADRC [15]. sLOAD = Sporadic Late Onset Alzheimer Disease. CU = Cognitively Unimpaired. CI = Cognitively Impaired. CDR = Clinical Dementia Rating. *APOE* = Apolipoprotein E. BAG = Brain Age Gap from DeepBrainNet [20].
